# Resource prioritization and balancing for the quantum internet

**DOI:** 10.1038/s41598-020-78960-5

**Published:** 2020-12-28

**Authors:** Laszlo Gyongyosi, Sandor Imre

**Affiliations:** 1 Department of Networked Systems and Services, Budapest University of Technology and Economics, Budapest, 1117 Hungary; 2 MTA-BME Information Systems Research Group, Hungarian Academy of Sciences, Budapest, 1051 Hungary

**Keywords:** Mathematics and computing, Computer science, Pure mathematics

## Abstract

The quantum Internet enables networking based on the fundamentals of quantum mechanics. Here, methods and procedures of resource prioritization and resource balancing are defined for the quantum Internet. We define a model for resource consumption optimization in quantum repeaters, and a strongly-entangled network structure for resource balancing. We study the resource-balancing efficiency of the strongly-entangled structure. We prove that a strongly-entangled quantum network is two times more efficient in a resource balancing problem than a full-mesh network of the traditional Internet.

## Introduction

The quantum Internet^1–30^ aims to provide an adequate answer to the computational power that becomes available with quantum computers^31–60^. To provide a seamless transition to the legal users from the traditional Internet to the quantum Internet, the creation of advanced services and methods for the quantum Internet are emerging tasks^52–54,61–67^. The quantum Internet is modeled as a quantum network consisting of quantum repeaters and entangled connections between the quantum repeaters^2,66–129^. This entangled quantum network forms a general framework for the quantum Internet, enabling long-distance quantum communications, multi-hop entanglement and multi-hop QKD (quantum key distribution)^25^, utilization of quantum protocols, advanced distributed computing, high-precision sensor networks, and the establishment of a global-scale quantum Internet.

A crucial problem related to the quantum Internet is the resource optimization of the quantum repeaters and the handling of resource requirement issues such as non-servable resource requests in the quantum repeaters^18–26^. These fundamental questions are still open and have not been addressed for the quantum Internet.

Here, we define methods for resource prioritization and resource balancing for the quantum Internet. The aim of the proposed solutions is to optimize the resource allocation mechanisms and to reduce the resource consumption of the network entities of the quantum Internet. A model of resource consumption^130–134^ of quantum repeaters is proposed, and its optimization is realized through the weightings of the entanglement throughputs of the entangled connections of the quantum repeaters. We also propose a method for optimizing the entanglement swapping procedure and determine the conditions of deadlock-free entanglement swapping. For resource balancing, a strongly-entangled network structure is defined. This network is modeled as an independent entity in the quantum Internet, composed of an arbitrary number of quantum repeaters such that all quantum repeaters are entangled with each other. The primary aim of the strongly-entangled structure is to serve those quantum nodes that have non-servable resource requests due to resource issues or an arbitrary network issue; these quantum nodes are referred to as low-priority quantum nodes.

The strongly-entangled structure injects additional resources into the quantum network to manage the resource issues of an arbitrary number of low-priority quantum nodes. The structure also provides optimized resource balancing for the low-priority quantum nodes. We prove the resource-balancing efficiency of the strongly-entangled structure and study its fault tolerance. We show that a strongly-entangled quantum network structure, due to the advanced attributes of quantum networking, is two times more efficient in resource balancing than a classical full-mesh^135,136^ network structure.

The novel contributions of our manuscript are as follows:

1. We define methods and procedures for resource prioritization and resource balancing in the quantum Internet.
2. The resource prioritization covers the resource consumption optimization of the quantum repeaters via the entanglement throughput weightings, prioritization of entanglement swapping in the quantum repeaters, and deadlock-free entanglement swapping.
3. A strongly-entangled structure is defined for an optimal resource balancing. We prove the resource-balancing efficiency of the proposed structure and prove its fault tolerance. We show that a strongly-entangled quantum network structure is two times more efficient in resource balancing than a classical full-mesh network structure.

This paper is organized as follows. In “Preliminaries” section, preliminaries are summarized. In “Method” section, methods for resource consumption optimization are defined. “Strongly-entangled structure for resource balancing in the quantum internet” section proposes a solution for optimal resource balancing. A performance analysis is given in “Performance evaluation” section. Finally, “Conclusions” section provides the conclusions. Supplementary information is included in the Appendix.

## Preliminaries

### Basic terms

#### Entanglement fidelity

The aim of the entanglement distribution procedure is to establish a *d*-dimensional entangled system between the distant points *A* and *B*, through the intermediate quantum repeater nodes. Let $d=2$, and let ${\left| \beta _{00} \right\rangle } =\frac{1}{\sqrt{2} } \left( {\left| 00 \right\rangle } +{\left| 11 \right\rangle } \right)$ be the entangled state subject to be established between distant points *A* and *B*. At a particular two-partite state $\sigma$ established between *A* and *B*, the fidelity of $\sigma$ is evaluated as

1 $$\begin{aligned} F=\left\langle {{\beta }_{00}} | \sigma |{{\beta }_{00}} \right\rangle . \end{aligned}$$

Without loss of generality, an aim of a practical entanglement distribution is to reach $F\ge 0.98$^2–4,12,68,69,137,138^.

#### Entangled network structure

Let *V* refer to the nodes of an entangled quantum network *N*, which consists of a transmitter node $$A\in V$$, a receiver node $$B\in V$$, and quantum repeater nodes $$R_{i} \in V$$, $$i=1,\ldots ,q$$. Let $$E=\left\{ E_{j} \right\} $$, $$j=1,\ldots ,m$$ refer to a set of edges (an edge refers to an entangled connection in a graph representation) between the nodes of *V*, where each $$E_{j} $$ identifies an $${\mathrm{L}}_{l} $$-level entanglement, $$l=1,\ldots ,r$$, between quantum nodes $$x_{j} $$ and $$y_{j} $$ of edge $$E_{j} $$, respectively. Let $$N=\left( V,{{\mathscr {S}}}\right) $$ be an actual quantum network with $$\left| V\right| $$ nodes and a set $${{\mathscr {S}}}$$ of entangled connections. An $${\mathrm{L}}_{l} $$-level, $$l=1,\ldots ,r$$, entangled connection $$E_{{\mathrm{L}}_{l} } \left( x,y\right) $$, refers to the shared entanglement between a source node *x* and a target node *y*, with hop-distance

2 $$\begin{aligned} d\left( x,y\right) _{{\mathrm{L}}_{l} } =2^{l-1} , \end{aligned}$$

since the entanglement swapping (extension) procedure doubles the span of the entangled pair in each step. This architecture is also referred to as the doubling architecture^2,68,69,138^.

For a particular ${\mathrm{L}}_{l}$-level entangled connection $E_{{\mathrm{L}}_{l} } \left( x,y\right)$ with hop-distance (2), there are $d\left( x,y\right) _{{\mathrm{L}}_{l} } -1$ intermediate nodes between the quantum nodes *x* and *y*.

### Entanglement throughput

Let $B_{F} (E_{{\mathrm{L}}_{l} }^{i})$ refer to the entanglement throughput of a given ${\mathrm{L}}_{l}$ entangled connection $E_{{\mathrm{L}}_{l} }^{i}$ measured in the number of *d*-dimensional entangled states established over $E_{{\mathrm{L}}_{l} }^{i}$ per sec at a particular fidelity *F* (dimension of a qubit system is $d=2$)^2–4,12,68,69,137,138^.

For any entangled connection $E_{{\mathrm{L}}_{l} }^{i}$, a condition *c* should be satisfied, as

3 $$\begin{aligned} c:{{B}_{F}}( E_{{{\text {L}}_{l}}}^{i})\ge {B}_{F}^{\text {*}}( E_{{{\text {L}}_{l}}}^{i}),\text { for }\forall i, \end{aligned}$$

where ${{B}}_{F}^{\text {*}}( E_{{{\text {L}}_{l}}}^{i})$ is a critical lower bound on the entanglement throughput at a particular fidelity *F* of a given $E_{{{\text {L}}_{l}}}^{i}$, i.e., ${{B}_{F}}( E_{{{\text {L}}_{l}}}^{i})$ of a particular $E_{{{\text {L}}_{l}}}^{i}$ has to be at least ${B}_{F}^{\text {*}}( E_{{{\text {L}}_{l}}}^{i})$.

#### Oscillator cycles

To quantify the entanglement throughput of the entangled connections, time is measured in number of cycles *C*. The time $$t_{C} $$ of a cycle *C* is determined by an oscillator unit $$O_{C} $$ that is available for all the entities of the quantum network, such that $${{t}_{C}}={1}/{{{f}_{C}}}$$, where $$f_{C} $$ is the frequency of $$O_{C} $$, with $${{f}_{C}}={1}/{{{t}_{C}}}$$.

### Definitions

#### Resource consumption of a quantum repeater

Let $$\alpha \left( R_{i} ,{\mathrm{L}}_{l} \left( k\right) \right) $$ be the resource consumption of quantum repeater $$R_{i} $$ associated with a *k*-th entangled connection $${\mathrm{L}}_{l} \left( k\right) $$, $$k=1,\ldots ,z$$, where *l* is the level of entanglement of the connection and *z* is the total number of entangled connections of $$R_{i} $$.

Let $$\Upsilon \left( R_{i} ,{\mathrm{L}}_{l} \left( k\right) \right) $$ be the resource consumption of quantum repeater $$R_{i} $$ associated with the quantum memory usage at $${\mathrm{L}}_{l} \left( k\right) $$; let $$\phi \left( R_{i} ,{\mathrm{L}}_{l} \left( k\right) \right) $$ be the resource consumption of quantum repeater $$R_{i} $$ associated with the entanglement purification of $${\mathrm{L}}_{l} \left( k\right) $$; let $$\tau \left( R_{i} ,{\mathrm{L}}_{l} \left( k\right) \right) $$ be the resource consumption of quantum repeater $$R_{i} $$ associated with the entanglement distribution to a target node *B*; and let $$\nu \left( R_{i} ,{\mathrm{L}}_{l} \left( k\right) \right) $$ be the resource consumption of quantum repeater $$R_{i} $$ associated with the entanglement swapping $$U_{S} $$ of $${\mathrm{L}}_{l} \left( k\right) $$. Then, $$\alpha \left(R_{i} ,{\mathrm{L}}_{l} \left( k\right) \right) $$ can be defined as

4 $$\begin{aligned} \alpha \left( R_{i} ,{\mathrm{L}}_{l} \left( k\right) \right) :=B_{F} \left( {\mathrm{L}}_{l} \left( k\right) \right) \left( \partial \left( R_{i} ,{\mathrm{L}}_{l} \left( k\right) \right) \right) +\zeta \left( R_{i} ,{\mathrm{L}}_{l} \left( k\right) \right) +C\left( R_{i} ,{\mathrm{L}}_{l} \left( k\right) \right) , \end{aligned}$$

where the term $\partial \left( R_{i} ,{\mathrm{L}}_{l} \left( k\right) \right)$ is defined as

5 $$\begin{aligned} \partial \left( R_{i} ,{\mathrm{L}}_{l} \left( k\right) \right) := \Upsilon \left( R_{i} ,{\mathrm{L}}_{l} \left( k\right) \right) +\phi \left( R_{i} ,{\mathrm{L}}_{l} \left( k\right) \right) +\tau \left( R_{i} ,{\mathrm{L}}_{l} \left( k\right) \right) +\nu \left( R_{i} ,{\mathrm{L}}_{l} \left( k\right) \right) , \end{aligned}$$

where $${B_{F} \left( {\mathrm{L}}_{l} \left( k\right) \right)} $$ is the entanglement throughput (Bell states per *C*) of the entangled connection $${{\mathrm{L}}_{l} \left( k\right)} $$, while $${\zeta \left( R_{i} ,{\mathrm{L}}_{l} \left( k\right) \right)} $$ identifies the resource consumption of quantum repeater $$R_{i} $$ associated with the path selection, and $$C\left( R_{i} ,{\mathrm{L}}_{l} \left( k\right) \right) $$ refers to the cost of auxiliary classical communications.

#### Set of outcoming entangled states

Let $$\rho _{A} $$ be an input entangled density matrix (i.e., half pair of an entangled state) in quantum repeater $$R_{i} $$, and let $${{\mathscr {A}}}\left( \rho _{A} \right) $$ be the set of possible *r* outcoming entangled states in $$R_{i} $$,

6 $$\begin{aligned} {{\mathscr {A}}}\left( \rho _{A} \right) :=\left\{ {{\sigma }_{B,1}},\ldots ,{{\sigma }_{B,r}} \right\} , \end{aligned}$$

where $$\sigma _{B,i} $$ is the *i*-th possible outcoming density matrix. The set $${{\mathscr {A}}}\left( \rho _{A} \right) $$ is therefore identifies those (purified) entangled states, that can be selected for the $$U_{S} $$ entanglement swapping with $$\rho _{A} $$ to formulate an extended entangled connection via $$R_{i} $$.

#### Extended entangled connection

Using (6), an extended entangled connection is depicted as

7 $$\begin{aligned} {\mathrm{L}}_{l} \left( R_{s} \left( \beta \left( \rho_{A} \right) \right) ,R_{d} \left( \beta \left( \sigma_{B} \right) \right) \right) , \end{aligned}$$

where $$\beta \left( \rho _{A} \right) $$ identifies subsystem $$\rho _{A} $$ of the entangled state $$\beta _{AB} $$, $$\beta \left( \sigma_{B} \right) $$ identifies subsystem $$\sigma _{B} $$ of the entangled state $$\beta _{AB} $$, $$R_{s} \left( \beta \left( \rho _{A} \right) \right) $$ is the $$R_{s} $$ source quantum node with $$\beta \left( \rho _{A} \right) $$, while $$R_{d} \left( \beta \left( \sigma _{B} \right) \right) $$ is the $$R_{d} $$ destination quantum node with $$\beta \left( \sigma _{B} \right) $$, where $$\beta \left( \sigma _{B} \right) $$ is selected from set $${{\mathscr {A}}}\left( \rho _{A} \right) $$ for the entanglement swapping to formulate $$\beta _{AB} $$.

#### Set of destination quantum nodes

The set $${{\mathscr {A}}}\left( \rho _{A} \right) $$ in (6) is determined for a particular incoming density $$\rho _{A} $$ by the set $${{{\mathscr {D}}}}\left( R_{i} \right) $$ of $$R_{d} $$ destination quantum nodes that share an entangled connection with a current quantum repeater $$R_{i} $$, as

8 $$\begin{aligned} {{{\mathscr {D}}}}\left( R_{i} \right) :=\left\{ {{R}_{d}}\left( \beta \left( {{\sigma }_{B,1}} \right) \right) ,\ldots ,{{R}_{d}}\left( \beta \left( {{\sigma }_{B,r}} \right) \right) \right\} , \end{aligned}$$

where $$R_{d} \left( \beta \left( \sigma _{B,i} \right) \right) $$ refers to the $$R_{d} $$ destination quantum node with $$\beta \left( \sigma _{B,i} \right) $$, $$i=1,\ldots ,r$$.

#### Set of entangled connections via swapping

Let $${{\mathscr {S}}}_{{\mathscr {P}}} \left( R_{i} ,\rho _{A} \right) $$ refer to the set of entangled connections that contains the entangled connection that is resulted between distant source $$R_{s} $$ and destination $$R_{d} $$ via an entanglement swapping in a particular quantum repeater $$R_{i} $$ using input state $$\rho _{A} $$ and output state $$\sigma _{B} $$, as

9 $$\begin{aligned} {{\mathscr {S}}}_{{\mathscr {P}}} \left( R_{i} ,\rho _{A} \right) :={{\mathscr {S}}}_{{\mathscr {P}}} \left( \sigma _{B} ,R_{s} \left( \beta \left( \rho _{A} \right) \right) ,R_{d} \left( \beta \left( \sigma _{B} \right) \right) \right) \subseteq {{\mathscr {S}}}_{{\mathscr {P}}} \left( R_{i} ,R_{d} \left( \beta \left( \sigma _{B} \right) \right) \right) , \end{aligned}$$

where $${{\mathscr {S}}}_{{\mathscr {P}}} \left( R_{i} ,R_{d} \left( \sigma _{B} \right) \right) $$ is the set of paths that pass through $$R_{i} $$ using the entangled pair $$\beta \left( \sigma _{B} \right) $$ in $$R_{d} $$.

#### Strongly-entangled structure

For a $${{\mathscr {S}}}_{{\mathscr {N}}} $$ strongly-entangled structure, the number of low-priority quantum nodes (quantum nodes with non-servable resource requests) in *N* is $$n_{c} $$, while $$\left| {{\mathscr {S}}}_{{\mathscr {N}}} \right| $$ is the number of quantum repeaters in a $${{\mathscr {S}}}_{{\mathscr {N}}} $$ strongly-entangled structure, $$R_{1}^{\left( {{\mathscr {S}}}_{{\mathscr {N}}} \right) } ,\ldots ,R_{\left| {{\mathscr {S}}}_{{\mathscr {N}}} \right| }^{\left( {{\mathscr {S}}}_{{\mathscr {N}}} \right) } .$$ Since $${{\mathscr {S}}}_{{\mathscr {N}}} $$ is strongly-entangled, each quantum repeater in $${{\mathscr {S}}}_{{\mathscr {N}}} $$ has $$\left| {{\mathscr {S}}}_{{\mathscr {N}}} \right| -1$$ entangled connections, and the $$\left| E\left( {{\mathscr {S}}}_{{\mathscr {N}}} \right) \right| $$ number of entangled connections within $${{\mathscr {S}}}_{{\mathscr {N}}} $$ is

10 $$\begin{aligned} \left| E\left( {{\mathscr {S}}}_{{\mathscr {N}}} \right) \right| :={\textstyle \frac{\left| {{\mathscr {S}}}_{{\mathscr {N}}} \right| \cdot \left( \left| {{\mathscr {S}}}_{{\mathscr {N}}} \right| -1\right) }{2}} . \end{aligned}$$

The entanglement levels of the $\left| E\left( {{\mathscr {S}}}_{{\mathscr {N}}} \right) \right|$ entangled connections in ${{\mathscr {S}}}_{{\mathscr {N}}}$ are defined in the following manner. Let *A* be the ingress node of ${{\mathscr {S}}}_{{\mathscr {N}}}$, and let *B* be the egress node of ${{\mathscr {S}}}_{{\mathscr {N}}}$, with hop-distance $d\left( A,B\right)$. Then, the $L\left( d(x,y\right) )$ entangled connections in function of the $d\left( x,y\right) )$ hop-distance between quantum nodes $\left\{ x,y\right\} \in {{\mathscr {S}}}_{{\mathscr {N}}}$ in the ${{\mathscr {S}}}_{{\mathscr {N}}}$ strongly-entangled structure are distributed as follows:

11 $$\begin{aligned} L(d\left( x,y\right) ):=\left\{ \begin{array}{l} {\left| L\left( 1\right) \right| =\left| {{\mathscr {S}}}_{{\mathscr {N}}} \right| -1=d\left( A,B\right) } \\ {\left| L\left( 2\right) \right| =\left| L\left( 1\right) \right| -1} \\ {\vdots } \\ {\left| L\left( \left| {{\mathscr {S}}}_{{\mathscr {N}}} \right| -2\right) \right| =\left| L\left( \left| {{\mathscr {S}}}_{{\mathscr {N}}} \right| -3\right) \right| -1} \\ {\left| L\left( \left| {{\mathscr {S}}}_{{\mathscr {N}}} \right| \right) -1\right| =L\left( d\left( A,B\right) \right) =1} \end{array}\right. , \end{aligned}$$

at a particular number $\left| {{\mathscr {S}}}_{{\mathscr {N}}} \right|$ of quantum nodes. (Note, the strongly-entangled structure utilizes different entanglement levels than the doubling architecture, therefore in (11) the entanglement levels are denoted in different manner.)

#### Capability of a strongly-entangled structure

Assuming that there is a set $${{\mathscr {S}}}_{n_{c} } $$ of $$n_{c} $$ low-priority $$R_{i} $$ quantum repeaters in the network, $$i=1,\ldots ,n_{c} $$, the $$R_{q}^{\left( {{\mathscr {S}}}_{{\mathscr {N}}} \right) } $$ in $${{\mathscr {S}}}_{{\mathscr {N}}} $$ is associated with entanglement throughput request (Bell states per *C*)

12 $$\begin{aligned} B\left( R_{q}^{\left( {{\mathscr {S}}}_{{\mathscr {N}}} \right) } ,{{\mathscr {S}}}_{n_{c} } \right) :={\textstyle \frac{1}{\left| {{\mathscr {S}}}_{{\mathscr {N}}} \right| }} \left( \sum _{i=1}^{n_{c} }B\left( R_{i} \right) \right) , \end{aligned}$$

while for the internal entangled connections

13 $$\begin{aligned} Q\left( R_{q}^{\left( {{\mathscr {S}}}_{{\mathscr {N}}} \right) } ,R_{z}^{\left( {{\mathscr {S}}}_{{\mathscr {N}}} \right) } \right) :={\textstyle \frac{1}{\left| {{\mathscr {S}}}_{{\mathscr {N}}} \right| ^{2} }} \left( \sum _{i=1}^{n_{c} }B\left( R_{i} \right) \right) . \end{aligned}$$

where $R_{z}^{\left( {{\mathscr {S}}}_{{\mathscr {N}}} \right) }$ is a neighbor of $R_{q}^{\left( {{\mathscr {S}}}_{{\mathscr {N}}} \right) }$ in ${{\mathscr {S}}}_{{\mathscr {N}}}$, $z\ne q$, $q=1,\ldots ,\left| {{\mathscr {S}}}_{{\mathscr {N}}} \right|$.

Since, by definition, $R_{q}^{\left( {{\mathscr {S}}}_{{\mathscr {N}}} \right) }$ has $\left| {{\mathscr {S}}}_{{\mathscr {N}}} \right| -1$ entangled connections in ${{\mathscr {S}}}_{{\mathscr {N}}}$, it follows that the $W\left( R_{q}^{\left( {{\mathscr {S}}}_{{\mathscr {N}}} \right) } \right)$ total entanglement throughput associated with $R_{q}^{\left( {{\mathscr {S}}}_{{\mathscr {N}}} \right) }$ within the structure of ${{\mathscr {S}}}_{{\mathscr {N}}}$ (Bell states per *C*) is as

14 $$\begin{aligned} \begin{aligned} W\left( R_{q}^{\left( {{\mathscr {S}}_{\mathscr {N}}} \right) } \right)&:=\sum \limits _{\left( q,z \right) :z\ne q}{Q\left( R_{q}^{\left( {{\mathscr {S}}_{\mathscr {N}}} \right) },R_{z}^{\left( {{\mathscr {S}}_{\mathscr {N}}} \right) } \right) } \\&=\left( \left| {{\mathscr {S}}_{\mathscr {N}}} \right| -1 \right) \tfrac{1}{\left| {{\mathscr {S}}_{\mathscr {N}}} \right| }B\left( R_{q}^{\left( {{\mathscr {S}}_{\mathscr {N}}} \right) },{{\mathscr {S}}_{{{n}_{c}}}} \right) \\&=\left( \left| {{\mathscr {S}}_{\mathscr {N}}} \right| -1 \right) \tfrac{1}{{{\left| {{\mathscr {S}}_{\mathscr {N}}} \right| }^{2}}}\left( \sum \limits _{i=1}^{{{n}_{c}}}{B\left( {{R}_{i}} \right) } \right) . \end{aligned} \end{aligned}$$

Since there are $\left| {{\mathscr {S}}}_{{\mathscr {N}}} \right|$ quantum repeaters in ${{\mathscr {S}}}_{{\mathscr {N}}}$, the $Z\left( {{\mathscr {S}}}_{{\mathscr {N}}} \right)$ cumulated entanglement throughput of the quantum repeaters of ${{\mathscr {S}}}_{{\mathscr {N}}}$ (Bell states per *C*) is as

15 $$\begin{aligned} \begin{aligned} Z\left( {{\mathscr {S}}_{\mathscr {N}}} \right)&:=\sum \limits _{q=1}^{\left| {{\mathscr {S}}_{\mathscr {N}}} \right| }{W\left( R_{q}^{\left( {{\mathscr {S}}_{\mathscr {N}}} \right) } \right) } \\&=\sum \limits _{q=1}^{\left| {{\mathscr {S}}_{\mathscr {N}}} \right| }{\sum \limits _{\left( q,z \right) :z\ne q}{Q\left( R_{q}^{\left( {{\mathscr {S}}_{\mathscr {N}}} \right) },R_{z}^{\left( {{\mathscr {S}}_{\mathscr {N}}} \right) } \right) }} \\&=\sum \limits _{q=1}^{\left| {{\mathscr {S}}_{\mathscr {N}}} \right| }{\left( \left| {{\mathscr {S}}_{\mathscr {N}}} \right| -1 \right) \tfrac{1}{\left| {{\mathscr {S}}_{\mathscr {N}}} \right| }B\left( R_{q}^{\left( {{\mathscr {S}}_{\mathscr {N}}} \right) },{{\mathscr {S}}_{{{n}_{c}}}} \right) } \\&=\left| {{\mathscr {S}}_{\mathscr {N}}} \right| \left( \left| {{\mathscr {S}}_{\mathscr {N}}} \right| -1 \right) \tfrac{1}{\left| {{\mathscr {S}}_{\mathscr {N}}} \right| }B\left( R_{q}^{\left( {{\mathscr {S}}_{\mathscr {N}}} \right) },{{\mathscr {S}}_{{{n}_{c}}}} \right) \\&=\left| {{\mathscr {S}}_{\mathscr {N}}} \right| \left( \left| {{\mathscr {S}}_{\mathscr {N}}} \right| -1 \right) \tfrac{1}{{{\left| {{\mathscr {S}}_{\mathscr {N}}} \right| }^{2}}}\left( \sum \limits _{i=1}^{{{n}_{c}}}{B\left( {{R}_{i}} \right) } \right) \\&=\left( \left| {{\mathscr {S}}_{\mathscr {N}}} \right| -1 \right) \tfrac{1}{\left| {{\mathscr {S}}_{\mathscr {N}}} \right| }\left( \sum \limits _{i=1}^{{{n}_{c}}}{B\left( {{R}_{i}} \right) } \right) . \end{aligned} \end{aligned}$$

Because of the ${{\mathscr {S}}}_{{\mathscr {N}}}$ strongly-entangled structure has ${\left| {{\mathscr {S}}}_{{\mathscr {N}}} \right| \left( \left| {{\mathscr {S}}}_{{\mathscr {N}}} \right| -1\right) \big / 2}$ entangled connections, the $T\left( {{\mathscr {S}}}_{{\mathscr {N}}} \right)$ total entanglement throughput of the entangled connections of ${{\mathscr {S}}}_{{\mathscr {N}}}$ (Bell states per *C*) is as

16 $$\begin{aligned} \begin{aligned} T\left( {{\mathscr {S}}_{\mathscr {N}}} \right)&:=\left| {{\mathscr {S}}_{\mathscr {N}}} \right| \left( \tfrac{\left| {{\mathscr {S}}_{\mathscr {N}}} \right| -1}{2} \right) \tfrac{1}{{{\left| {{\mathscr {S}}_{\mathscr {N}}} \right| }^{2}}}\left( \sum \limits _{i=1}^{{{n}_{c}}}{B\left( {{R}_{i}} \right) } \right) \\&=\left( \tfrac{\left| {{\mathscr {S}}_{\mathscr {N}}} \right| -1}{2} \right) \tfrac{1}{\left| {{\mathscr {S}}_{\mathscr {N}}} \right| }\left( \sum \limits _{i=1}^{{{n}_{c}}}{B\left( {{R}_{i}} \right) } \right) . \end{aligned} \end{aligned}$$

## Related works

In this section the related works are given.

On the problem of resource allocation and routing in quantum networks, we suggest the works of^62,70,71^. In^70^, the authors study the problem of entanglement routing in practical quantum networks with limited quantum processing capabilities and with noisy optical links. The authors study how a practical quantum network can distribute high-rate entanglement simultaneously between multiple pairs of users. In^71^, the authors study new routing algorithms for a quantum network with noisy quantum devices such that each can store a small number of qubits. In^62^, the problem of entanglement generation is modeled through a stochastic framework that takes into consideration the key physical-layer mechanisms affecting the end-to-end entanglement rate. The author derives the closed-form expression of the end-to-end entanglement rate for an arbitrary path, and design a routing protocol for quantum networks.

In a quantum Internet scenario, the entanglement purification is a procedure that takes two imperfect systems $\sigma _{1}$ and $\sigma _{2}$ with initial fidelity $F_0<1$, and outputs a higher-fidelity density $\rho$ such that $F\left( \rho \right) >F_0$. In^139^, the authors propose novel physical approaches to assess and optimize entanglement purification schemes. The proposed solutions provide an optimization framework of practical entanglement purification.

In^140^, a satellite-to-ground QKD system has been demonstrated. In^141^, the authors demonstrated the quantum teleportation of independent single-photon qubits. In^142^, the authors demonstrated the Bell inequality violation using electron spins. In^143^, the authors demonstrated modular entanglement of atomic qubits using photons and phonons. For an experimental realization of quantum repeaters based on atomic ensembles and linear optics, see^144,145^.

Since quantum channels also have a fundamental role in the quantum Internet, we suggest the review paper of^137^, for some specialized applications of quantum channels. For a review on some recent results of quantum computing technology, we suggest^146^. For some recent services developed for the quantum Internet, we suggest^12–17,27–29^.

Some other related topics are as follows. The works^12–14,68,69,137,138^ are related to the utilization of entanglement for long-distance quantum communications and for a global-scale quantum Internet, and also to the various aspects of quantum networks in a quantum Internet setting^137,147–155^.

A technical roadmap on the experimental development of the quantum Internet has been provided in^20^, see also^156^. For some important works on the experimental implementations, we suggest^157–180^.

## Method

### Resource consumption optimization via entanglement throughput prioritization

The aim of the entanglement throughput prioritization is to find an optimal distribution of the entanglement throughputs of the entangled connections of a given quantum repeater. The prioritization leads to an optimized, nearly uniform distribution of the resource consumptions of the quantum repeaters.

#### Theorem 1

(Resource consumption of a quantum repeater). *The* $${{{\mathscr {C}}}}\left( R_{i} \right) $$ *resource consumption of a quantum repeater* $$R_{i} $$ *is adjustable by distributing the weight coefficients associated with the entanglement throughputs of the entangled connections of* $$R_{i} $$.

#### *Proof*

Let us assume that there are a source node *A* and a destination node *B* in the network.

Assuming that the total number of the (logical) incoming entangled connections $${\mathrm{L}}_{l} \left( k\right) $$ of quantum repeater $$R_{i} $$ is *z*, the total resource consumption $${{{\mathscr {C}}}}\left( R_{i} \right) $$ of quantum repeater $$R_{i} $$ is defined via the terms of “Resource consumption of a quantum repeater” section, as

17 $$\begin{aligned} {{{\mathscr {C}}}}\left( R_{i} \right) :=\sum _{k=1}^{z}\alpha \left( R_{i} ,{\mathrm{L}}_{l} \left( k\right) \right) . \end{aligned}$$

Then, let $$\chi \left( R_{i} \right) $$ be the total number of received entangled states (number of Bell states) in $$R_{i} $$ per cycle:

18 $$\begin{aligned} \chi \left( R_{i} \right) =\sum _{k=1}^{z}B_{F} \left( {\mathrm{L}}_{l} \left( k\right) \right) , \end{aligned}$$

which can be rewritten as a multiplication of the $$\left| B_{F} \left( A\right) \right| $$ number of entangled states outputted by a source node *A* to path $${{\mathscr {P}}}_{s} $$, and a $$\omega \left( {{\mathscr {P}}}_{s} \right) \in \left[ 0,1\right] $$ weight of an *s*-th path $${{\mathscr {P}}}_{s} $$, taken for all paths that pass through quantum repeater $$R_{i} $$ between *A* and *B*, as

19 $$\begin{aligned} \chi \left( R_{i} \right) =\sum _{\forall {{\mathscr {P}}}_{s} \left( A,B\right) \in {{\mathscr {S}}}_{{\mathscr {P}}} \left( R_{i} \right) }\omega \left( {{\mathscr {P}}}_{s} \left( A,B\right) \right) \left| B_{F} \left( {{\mathscr {P}}}_{s} \left( A\right) \right) \right| , \end{aligned}$$

where *A* and *B* are the source and target nodes associated with path $${{\mathscr {P}}}_{s} $$; $${{\mathscr {S}}}_{{\mathscr {P}}} \left( R_{i} \right) $$ is the set of $${{\mathscr {P}}}$$ paths that pass through quantum repeater $$R_{i} $$ between *A* and *B*, defined as

20 $$\begin{aligned} {{\mathscr {S}}}_{{\mathscr {P}}} \left( R_{i} \right) :=\left\{ \left. {{\mathscr {P}}}_{s} \left( x,y\right) \right| R_{i} \in {{\mathscr {P}}}_{s} \left( x,y\right) \right\} , \end{aligned}$$

with relation

21 $$\begin{aligned} \left| {{\mathscr {S}}}_{{\mathscr {P}}} \left( R_{i} ,A,B\right) \right| \le z; \end{aligned}$$

where ${{\mathscr {P}}}_{s} \left( x,y\right)$ is a *s*-th path between quantum nodes *x* and *y*, $s=1,\ldots ,\left| {{\mathscr {S}}}_{xy} \right|$, where ${{\mathscr {S}}}_{xy}$ is the set of paths between *x* and *y* and $\left| {{\mathscr {S}}}_{xy} \right|$ is the cardinality of ${{\mathscr {S}}}_{xy}$; such that for a given source and target pair $\left( A,B\right)$ of ${{\mathscr {P}}}_{s}$, $s=1,\ldots ,\left| {{\mathscr {S}}}_{AB} \right|$,

22 $$\begin{aligned} \sum _{s=1}^{\left| {{\mathscr {S}}}_{AB} \right| }\omega \left( {{\mathscr {P}}}_{s} \left( A,B\right) \right) =1. \end{aligned}$$

Using (18), the term in (17) can be rewritten as

23 $$\begin{aligned} \begin{aligned} \mathscr {C}\left( {{R}_{i}} \right)&\le \chi \left( {{R}_{i}} \right) \sum \limits _{k=1}^{z}{\left( \partial \left( {{R}_{i}},{{\text {L}}_{l}}\left( k \right) \right) \right) +\zeta \left( {{R}_{i}},{{\text {L}}_{l}}\left( k \right) \right) +C\left( {{R}_{i}},{{\text {L}}_{l}}\left( k \right) \right) } \\&=\sum \limits _{\forall {{\mathscr {P}}_{s}}\left( A,B \right) \in {{\mathscr {S}}_{\mathscr {P}}}\left( {{R}_{i}} \right) }{\sum \limits _{k=1}^{z}{\omega \left( {{\mathscr {P}}_{s}}\left( A,B \right) \right) |{{B}_{F}}\left( {{\mathscr {P}}_{s}}\left( A \right) \right) |\left( \partial \left( {{R}_{i}},{{\text {L}}_{l}}\left( k \right) \right) \right) +\zeta \left( {{R}_{i}},{{\text {L}}_{l}}\left( k \right) \right) +C\left( {{R}_{i}},{{\text {L}}_{l}}\left( k \right) \right) }}. \end{aligned} \end{aligned}$$

The result in (23) reveals that a loose upper bound on $\mathscr {C}\left( {{R}_{i}}\right)$ can be obtained from (17) and (18), and also shows that ${{{\mathscr {C}}}}\left( R_{i} \right)$ is adjustable by the weight coefficients $\omega \left( {{\mathscr {P}}}_{s} \right)$. An aim here is therefore to find the optimal distribution of the weight coefficients.

Assuming that the total number of quantum repeaters is *q*, the optimization problem can be defined via an objective function $f\left( {{{\mathscr {C}}}}\right)$ subject to a minimization as

24 $$\begin{aligned} f\left( {{{\mathscr {C}}}}\right) :=\min \left( \tilde{{\mathscr {C}}}\left( R_{i} \right) \right) ,{\mathrm{\; for\; }}1\le i\le q, \end{aligned}$$

where

25 $$\begin{aligned} \tilde{{\mathscr {C}}}\left( R_{i} \right) =\max \left( {{{\mathscr {C}}}}\left( R_{i} \right) \right) . \end{aligned}$$

The problem is therefore to find the optimal distribution for the weights of the paths associated with the entangled connections that minimizes the objective function (24).

Using (22), a constraint $$\Omega \left( R_{i} \right) $$ can be defined for all source and target node pairs $$\left( x,y\right) $$ that share an entangled connection $${\mathrm{L}}_{l} \left( x,y\right) $$ through $$R_{i} $$, as

26 $$\begin{aligned} \Omega \left( R_{i} \right) :=\sum _{s=1}^{\left| {{\mathscr {S}}}_{xy} \right| }\omega \left( {{\mathscr {P}}}_{s} \left( x,y\right) \right) =1,{\mathrm{\; for\; }}\forall {\mathrm{L}}_{l} \left( x,y\right) , \end{aligned}$$

where

27 $$\begin{aligned} 0\le \omega \left( {{\mathscr {P}}}_{s} \left( x,y\right) \right) \le 1. \end{aligned}$$

Then, let $$B_{F} \left( R_{i} ,R_{j} \right) $$ be the entanglement throughput (Bell states per *C*) between quantum repeaters $$R_{i} $$ and $$R_{j} $$ connected by the entangled connection $${\mathrm{L}}_{l} \left( R_{i} ,R_{j} \right) $$, with an upper bound $$B_{F}^{*} \left( R_{i} ,R_{j} \right) $$.

Using (19), a constraint $$\Gamma \left( {\mathrm{L}}_{l} \left( R_{i} ,R_{j} \right) \right) $$ can be defined for the $${{\mathscr {P}}}$$ paths that traverse an entangled connection $${\mathrm{L}}_{l} \left( R_{i} ,R_{j} \right) $$ between quantum repeaters $$R_{i} $$ and $$R_{j} $$ (see Fig. 2a), as

28 $$\begin{aligned} \Gamma \left( {\mathrm{L}}_{l} \left( R_{i} ,R_{j} \right) \right) :=\sum _{\forall {{\mathscr {P}}}_{s} \left( A,B\right) \in {{\mathscr {S}}}_{{\mathscr {P}}}^{{\mathrm{L}}_{l} \left( R_{i} ,R_{j} \right) } \left( R_{i} \right) \bigcap {{\mathscr {S}}}_{{\mathscr {P}}}^{{\mathrm{L}}_{l} \left( R_{i} ,R_{j} \right) } \left( R_{j} \right) }\omega \left( {{\mathscr {P}}}_{s} \left( A,B\right) \right) |B_{F} \left( {{\mathscr {P}}}_{s} \left( A\right) \right) |\le B_{F}^{*} \left( R_{i} ,R_{j} \right) , \end{aligned}$$

where $${{\mathscr {S}}}_{{\mathscr {P}}}^{{\mathrm{L}}_{l} \left( R_{i} ,R_{j} \right) } \left( R_{i} \right) \bigcap {{\mathscr {S}}}_{{\mathscr {P}}}^{{\mathrm{L}}_{l} \left( R_{i} ,R_{j} \right) } \left( R_{j} \right) $$ refers to the set of paths that pass through the entangled connection $${\mathrm{L}}_{l} \left( R_{i} ,R_{j} \right) $$ between quantum repeaters $$R_{i} $$ and $$R_{j} $$, respectively. As follows, in (28), a particular path

29 $$\begin{aligned} {{\mathscr {P}}}_{s} \left( A,B\right) ={\mathrm{L}}_{l} \left( A,B\right) \end{aligned}$$

traverses the entangled connection ${\mathrm{L}}_{l} \left( R_{i} ,R_{j} \right)$, if only the relation

30 $$\begin{aligned} {{\mathscr {P}}}_{s} \left( A,B\right) \in {{\mathscr {S}}}_{{\mathscr {P}}}^{{\mathrm{L}}_{l} \left( R_{i} ,R_{j} \right) } \left( R_{i} \right) \bigcap {{\mathscr {S}}}_{{\mathscr {P}}}^{{\mathrm{L}}_{l} \left( R_{i} ,R_{j} \right) } \left( R_{j} \right) \end{aligned}$$

holds.

Then, let ${{\mathscr {S}}}_{{\mathscr {P}}} \left( N\right) =\left\{ {{\mathscr {P}}}_{1} ,\ldots ,{{\mathscr {P}}}_{n} \right\}$ be the set of entangled paths, where ${{\mathscr {P}}}_{i}$ is an *i*-th entangled path, with a weighted entanglement throughput $\phi \left( {{\mathscr {P}}}_{i} \right)$ of the path ${{\mathscr {P}}}_{i}$ (Bell states per *C*), as

31 $$\begin{aligned} \phi \left( {{\mathscr {P}}}_{i} \right) =\omega \left( {{\mathscr {P}}}_{i} \left( A,B\right) \right) B_{F} \left( {{\mathscr {P}}}_{i} \left( A\right) \right) , \end{aligned}$$

where *A* is the source node of entangled path ${{\mathscr {P}}}_{i}$, $\omega \left( {{\mathscr {P}}}_{i} \left( A,B\right) \right)$ is the weight associated to ${{\mathscr {P}}}_{i} \left( A,B\right)$, while $B_{F} \left( {{\mathscr {P}}}_{i} \left( A\right) \right)$ is the entanglement throughput (Bell states per *C*) of the source node *A* of ${{\mathscr {P}}}_{i}$.

The optimal $D\left( \omega _{s} \left( x,y\right) \right)$ distribution of the weights that minimizes $f\left( {{{\mathscr {C}}}}\right)$ is determined via Procedure 1.

Procedure 1 assumes a quantum Internet scenario, in which a particular quantum repeater $$R_{i} $$ has several different (logical) incoming and (logical) outcoming entangled connections, and the number of paths that traverse a particular quantum repeater is distributed non-uniformly. $$\square $$


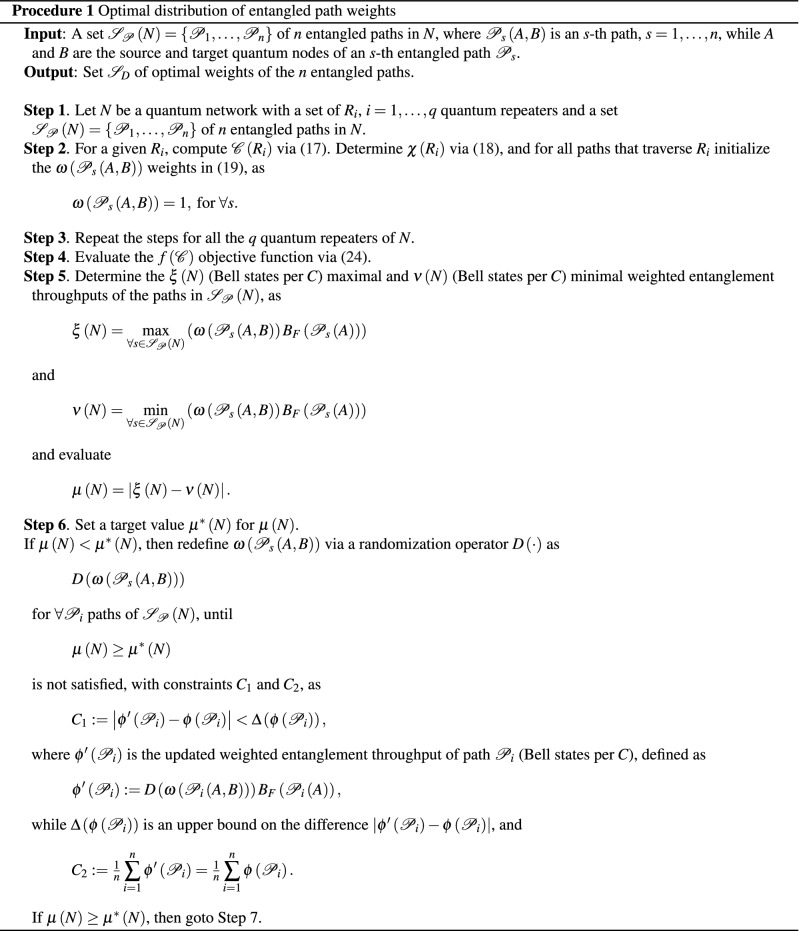

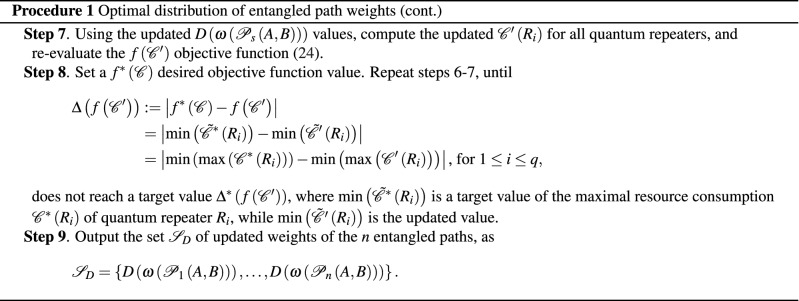




**Figure 1:**
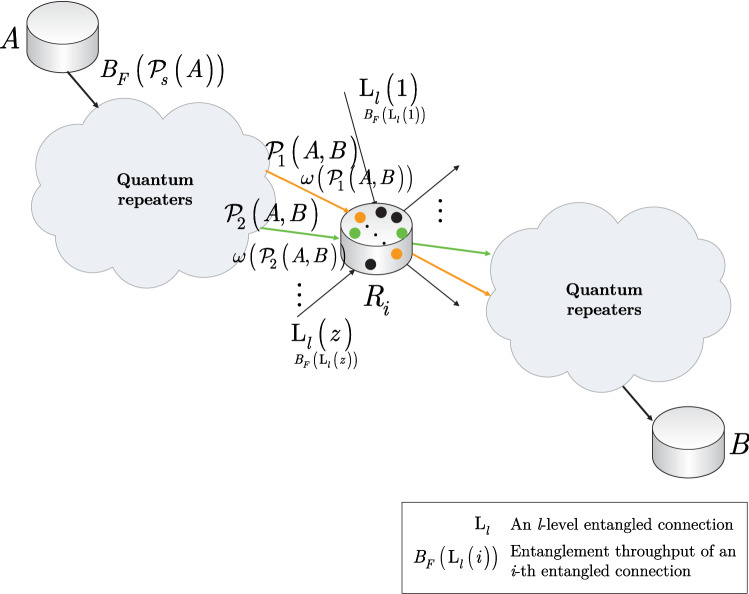
The schematic model of the resource consumption evaluation of a quantum repeater $$R_{i} $$ in a quantum Internet scenario. The quantum repeater has *z* incoming entangled connections, $${\mathrm{L}}_{l} \left( k\right) $$, $$k=1,\ldots ,z$$, from among $$\left| {{\mathscr {S}}}_{{\mathscr {P}}} \left( R_{i} \right) \right| =2$$ paths, $${{\mathscr {P}}}_{s} \left( A,B\right) $$, $$s=1,2$$, that pass through quantum repeater $$R_{i} $$ between *A* and *B*. The paths $${{\mathscr {P}}}_{1} \left( A,B\right) $$ and $${{\mathscr {P}}}_{2} \left( A,B\right) $$ are associated with the weighted entanglement throughput values $$\omega \left( {{\mathscr {P}}}_{1} \left( A,B\right) \right) B_{F} \left( {{\mathscr {P}}}_{s} \left( A\right) \right) $$ and $$\omega \left( {{\mathscr {P}}}_{2} \left( A,B\right) \right) B_{F} \left( {{\mathscr {P}}}_{s} \left( A\right) \right) $$, where $$\omega \left( {{\mathscr {P}}}_{s} \left( A,B\right) \right) \in \left[ 0,1\right] $$ are the path weights and $$B_{F} \left( {{\mathscr {P}}}_{s} \left( A\right) \right) $$ is the entanglement throughput (Bell states per *C*) of the source *A* of the path $${{\mathscr {P}}}_{s} $$. (The entangled states associated with the entangled connections in the quantum repeater are depicted by green, brown, and black dots.)

The schematic model of the resource consumption determination of a quantum repeater is depicted in Fig. 1.

### Entanglement swapping prioritization

Because the distribution of the weights $\omega \left( {{\mathscr {P}}}_{s} \left( A,B\right) \right)$ is determined via Procedure 1, the task in a given quantum repeater is then to determine the set of entangled states associated with the weighted entangled connections for the entanglement swapping procedure.

#### Lemma 1

(Entanglement swapping probability and the weights of entangled connections). *The probability* $$\Pr \left( U_{S} \left( \rho _{A} ,\sigma _{B,i} \right) \right) $$ *of entanglement swapping* $$U_{S} $$ *between a source* $$\rho _{A} $$ *and a target density matrix* $$\sigma _{B,i} $$ *in a quantum repeater depends on the weights associated with the swapped entangled connections.*

#### *Proof*

Let

$$\begin{aligned} \Pr \left( U_{S} \left( \rho _{A} ,\sigma _{B,i} \right) \right) =\Pr \left( \sigma _{B,i} ,R_{s} \left( \beta \left( \rho _{A} \right) \right) ,R_{d} \left( \beta \left( \sigma _{B,i} \right) \right) \right) \end{aligned}$$

be the probability that density $$\sigma _{B,i} $$ is selected from $${{\mathscr {A}}}\left( \rho _{A} \right) $$ to the entanglement swapping with $$\rho _{A} $$ by swapping operator $$U_{S} $$.

**Figure 2:**
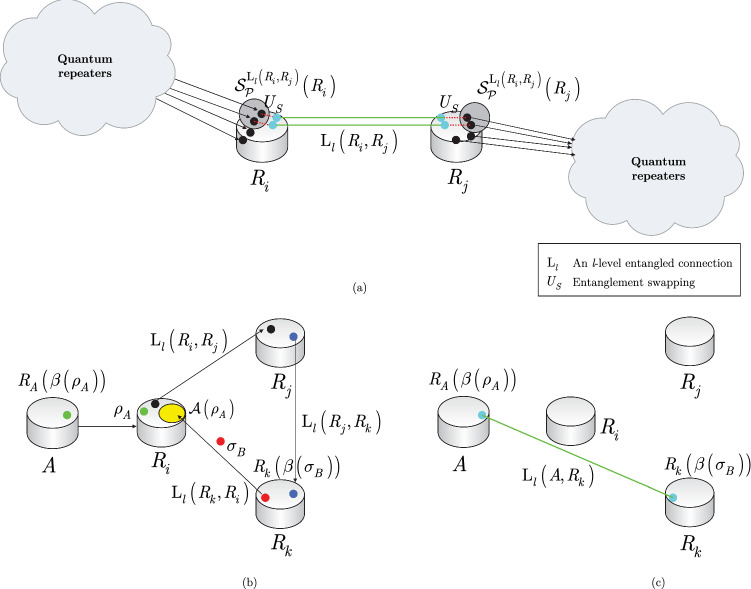
(**a**) A quantum Internet scenario with a set of incoming entangled connections $${{\mathscr {S}}}_{{\mathscr {P}}}^{{\mathrm{L}}_{l} \left( R_{i} ,R_{j} \right) } \left( R_{i} \right) \bigcap {{\mathscr {S}}}_{{\mathscr {P}}}^{{\mathrm{L}}_{l} \left( R_{i} ,R_{j} \right) } \left( R_{j} \right) $$ that traverse the entangled connection $${\mathrm{L}}_{l} \left( R_{i} ,R_{j} \right) $$ between quantum repeaters $$R_{i} $$ and $$R_{j} $$. The entangled states in the set $${{\mathscr {S}}}_{{\mathscr {P}}}^{{\mathrm{L}}_{l} \left( R_{i} ,R_{j} \right) } \left( R_{i} \right) $$ of $$R_{i} $$ and in the set $${{\mathscr {S}}}_{{\mathscr {P}}}^{{\mathrm{L}}_{l} \left( R_{i} ,R_{j} \right) } \left( R_{j} \right) $$ of $$R_{j} $$ (depicted by gray circles) are to be swapped with the entangled state that forms $${\mathrm{L}}_{l} \left( R_{i} ,R_{j} \right) $$. The entanglement swapping is performed by the entanglement swapping operator $$U_{S} $$. The other incoming entangled states in the quantum repeaters that do not traverse $${\mathrm{L}}_{l} \left( R_{i} ,R_{j} \right) $$ are not elements of $${{\mathscr {S}}}_{{\mathscr {P}}}^{{\mathrm{L}}_{l} \left( R_{i} ,R_{j} \right) } \left( R_{i} \right) \bigcap {{\mathscr {S}}}_{{\mathscr {P}}}^{{\mathrm{L}}_{l} \left( R_{i} ,R_{j} \right) } \left( R_{j} \right) $$. (**b**) A deadlock situation in the entanglement swapping procedure in a quantum Internet setting. The aim of quantum node *A* is to share an entangled connection with the distant quantum repeater $$R_{k} $$. The source node *A* generates an entangled pair and transmits one half, $$\rho _{A} $$, to $$R_{i} $$ and keeps the other half, $$R_{A} \left( \beta \left( \rho _{A} \right) \right) $$. In $$R_{i} $$, the set $${{\mathscr {A}}}\left( \rho _{A} \right) $$ (depicted by a yellow circle) does not contain the target entangled system $$\sigma _{B} $$ from the target node $$R_{k} $$ for the swapping; therefore, $$R_{i} $$ generates an entangled pair (depicted by black dots) and shares an entangled connection $${\mathrm{L}}_{l} \left( R_{i} ,R_{j} \right) $$ with $$R_{j} $$. Quantum repeater $$R_{j} $$ also generates an entangled pair (depicted by blue dots) and shares the entangled connection $${\mathrm{L}}_{l} \left( R_{j} ,R_{k} \right) $$ with $$R_{k} $$. Then, the target quantum node $$R_{k} $$ generates an entangled connection (depicted by red dots) and sends one half, $$\sigma _{B} $$, to $$R_{i} $$ to form the entangled connection $${\mathrm{L}}_{l} \left( R_{k} ,R_{i} \right) $$, while it keeps the other half, $$R_{k} \left( \beta \left( \sigma _{B} \right) \right) $$. (c) Quantum repeater $$R_{i} $$ receives $$\sigma _{B} $$ and swaps it with $$\rho _{A} $$ to form the distant entangled connection $${\mathrm{L}}_{l} \left( A,R_{k} \right) $$. The deadlock in the entanglement swapping is caused by the fact that set $${{\mathscr {A}}}\left( \rho _{A} \right) $$ in $$R_{i} $$ does not contain $$\sigma _{B} $$, so $$R_{i} $$ does not establish the entangled connection $${\mathrm{L}}_{l} \left( R_{i} ,R_{j} \right) $$ with $$R_{j} $$, and $$R_{j} $$ does not establish the entangled connection $${\mathrm{L}}_{l} \left( R_{j} ,R_{k} \right) $$ with $$R_{k} $$.

Since set ${{\mathscr {A}}}\left( \rho _{A} \right)$ contains *r* possible entangled states for the entanglement swapping,

43 $$\begin{aligned} \sum _{i=1}^{r}\Pr \left( U_{S} \left( \rho _{A} ,\sigma _{B,i} \right) \right) =\sum _{i=1}^{r}\Pr \left( \sigma _{B,i} ,R_{s} \left( \beta \left( \rho _{A} \right) \right) ,R_{d} \left( \beta \left( \sigma _{B,i} \right) \right) \right) =1, \end{aligned}$$

where probability $\Pr \left( \sigma _{B,i} ,R_{s} \left( \beta \left( \rho _{A} \right) \right) ,R_{d} \left( \beta \left( \sigma _{B,i} \right) \right) \right)$ is evaluated as

44 $$\begin{aligned} \begin{aligned} \Pr&\left( {{\sigma }_{B,i}},{{R}_{s}}\left( \beta \left( {{\rho }_{A}} \right) \right) ,{{R}_{d}}\left( \beta \left( {{\sigma }_{B,i}} \right) \right) \right) \\&=\sum \limits _{\forall {{\mathscr {P}}_{s}}\left( x,y \right) \in \kappa {{}_{\mathscr {P}}}}{{{\omega }_{s}}\left( {{\text {L}}_{l}}\left( {{R}_{s}}\left( \beta \left( {{\rho }_{A}} \right) \right) ,{{R}_{d}}\left( \beta \left( {{\sigma }_{B,i}} \right) \right) \right) \right) {{B}_{F}}\left( {{\text {L}}_{l}}\left( {{R}_{s}}\left( \beta \left( {{\rho }_{A}} \right) \right) ,{{R}_{d}}\left( \beta \left( {{\sigma }_{B,i}} \right) \right) \right) \right) ,} \end{aligned} \end{aligned}$$

where

45 $$\begin{aligned} \kappa _{{\mathscr {P}}} ={{\mathscr {S}}}_{{\mathscr {P}}} \left( \sigma _{B,i} ,R_{s} \left( \beta \left( \rho _{A} \right) \right) ,R_{d} \left( \beta \left( \sigma _{B,i} \right) \right) \right) , \end{aligned}$$

and $B_{F} \left( {\mathrm{L}}_{l} \left( R_{s} \left( \beta \left( \rho _{A} \right) \right) ,R_{d} \left( \beta \left( \sigma _{B,i} \right) \right) \right) \right)$ is the entanglement throughput (Bell states per *C*) of the entangled connection ${\mathrm{L}}_{l} \left( R_{s} \left( \beta \left( \rho _{A} \right) \right) ,R_{d} \left( \beta \left( \sigma _{B,i} \right) \right) \right)$, while $\omega _{s} \left( {\mathrm{L}}_{l} \left( R_{s} \left( \beta \left( \rho _{A} \right) \right) ,R_{d} \left( \beta \left( \sigma _{B,i} \right) \right) \right) \right)$ is the weight associated with an *s*-th path over ${\mathrm{L}}_{l} \left( R_{s} \left( \beta \left( \rho _{A} \right) \right) ,R_{d} \left( \beta \left( \sigma _{B,i} \right) \right) \right)$ (see also Fig. 2a).

Assuming that for each $\sigma _{B,i}$ there exist a source set ${{{\mathscr {Q}}}}\left( \sigma _{B,i} \right)$ of *g* input entangled states,

46 $$\begin{aligned} {{{\mathscr {Q}}}}\left( \sigma _{B,i} \right) =\left\{ {{\rho }_{A,1}},\ldots ,{{\rho }_{A,g}} \right\} , \end{aligned}$$

the probability $\Pr \left( \sigma _{B,i} ,{{{\mathscr {Q}}}}\left( \sigma _{B,i} \right) ,R_{d} \left( \beta \left( \sigma _{B,i} \right) \right) \right)$ can be yielded as

47 $$\begin{aligned} \begin{aligned} \Pr&\left( {{\sigma }_{B,i}},\mathscr {Q}\left( {{\sigma }_{B,i}} \right) ,{{R}_{d}}\left( \beta \left( {{\sigma }_{B}} \right) \right) \right) \\&=\sum \limits _{\forall {{\rho }_{A,k}}\in \mathscr {Q}\left( {{\sigma }_{B,i}} \right) }{\sum \limits _{\forall {{\mathscr {P}}_{s}}\left( x,y \right) \in \kappa {{}_{\mathscr {P},k}}}{{{\omega }_{s}}\left( {{\text {L}}_{l}}\left( {{R}_{s}}\left( \beta \left( {{\rho }_{A,k}} \right) \right) ,{{R}_{d}}\left( \beta \left( {{\sigma }_{B,i}} \right) \right) \right) \right) {{B}_{F}}\left( {{\text {L}}_{l}}\left( {{R}_{s}}\left( \beta \left( {{\rho }_{A,k}} \right) \right) ,{{R}_{d}}\left( \beta \left( {{\sigma }_{B,i}} \right) \right) \right) \right) ,}} \\ \end{aligned} \end{aligned}$$

where

48 $$\begin{aligned} \kappa _{{{\mathscr {P}}},k} ={{\mathscr {S}}}_{{\mathscr {P}}} \left( \sigma _{B,i} ,R_{s} \left( \beta \left( \rho _{A,k} \right) \right) ,R_{d} \left( \beta \left( \sigma _{B,i} \right) \right) \right) , \end{aligned}$$

and

49 $$\begin{aligned} \sum _{i=1}^{r}\Pr \left( \sigma _{B,i} ,{{{\mathscr {Q}}}}\left( \sigma _{B,i} \right) ,R_{d} \left( \beta \left( \sigma _{B,i} \right) \right) \right) =1. \end{aligned}$$

$$\square $$

#### Entanglement swapping deadlock

The entangled state selection procedure of the entanglement swapping in a quantum repeater $$R_{i} $$ can lead to a deadlock in the establishment of an entangled connection $$L_{l} \left( R_{i} ,R_{d} \right) $$ between $$R_{i} $$, and a distant quantum repeater $$R_{d} $$.

An entanglement swapping situation in a quantum Internet scenario is depicted in Fig. 2b, c, respectively.

The problem of deadlock-free entanglement swapping is discussed in Section A.1 of the Supplemental Information.

## Strongly-entangled structure for resource balancing in the quantum internet

A quantum network structure called the strongly-entangled quantum network is defined. The aim of this network is optimal resource balancing within the quantum Internet to take care of problematic situations. The problematic situation considered here is the serving of an arbitrary number of low-priority quantum nodes. A low-priority quantum node cannot be served by an actual quantum node in the network due to resource issues or an arbitrary network issue. Instead, the set of low-priority nodes are served through the strongly-entangled quantum network, which comprises an arbitrary number of quantum repeaters such that all quantum repeaters are entangled with each other. The strongly-entangled structure represents a resource that can manage issues in the network. In the serving procedure of the low-priority nodes, the quantum repeaters are selected uniformly at random to handle the density matrix of a low-priority node. The randomized behavior leads to a random routing between the low-priority nodes and the quantum repeaters, as well as to optimal resource balancing within the network. It is also assumed that the strongly-entangled structure has connections with many subnetworks.

### Resource allocation

In this section, the network situation is modeled via the definitions of “Strongly-entangled structure” section. A density matrix of $$R_{i} $$ is associated with an $$R_{I}^{\left( {{\mathscr {S}}}_{{\mathscr {N}}} \right) } $$ ingress quantum repeater of $${{\mathscr {S}}}_{{\mathscr {N}}} $$ selected uniformly at random, thus a random routing is performed for the incoming query from the low-priority node $$R_{i} $$ to $${{\mathscr {S}}}_{{\mathscr {N}}} $$. Then, an arbitrary routing is preformed between the $$R_{E}^{\left( {{\mathscr {S}}}_{{\mathscr {N}}} \right) } $$ egress quantum repeater of $${{\mathscr {S}}}_{{\mathscr {N}}} $$ and the $$D\left( R_{i} \right) $$ destination node of $$R_{i} $$.

The quantum nodes and the entangled connections of the $${{\mathscr {S}}}_{{\mathscr {N}}} $$ structure are characterized as follows. Let $$R_{q}^{\left( {{\mathscr {S}}}_{{\mathscr {N}}} \right) } $$ be an *q*-th, $$q=1,\ldots ,\left| {{\mathscr {S}}}_{{\mathscr {N}}} \right| $$, quantum repeater in $${{\mathscr {S}}}_{{\mathscr {N}}} $$, and let $$B\left( R_{i}^{\left( {{\mathscr {S}}}_{{\mathscr {N}}} \right) } ,R_{i} \right) $$ be the entanglement throughput request (Bell states per *C*) of the low-priority node $$R_{i} $$. The structure of a $${{\mathscr {S}}}_{{\mathscr {N}}} $$ strongly-entangled quantum network is depicted in Fig. 4.

#### Theorem 2

(Handling resource issues via a strongly-entangled structure). *Let* $$R_{i} $$ *be a low-priority quantum node with a non-servable resource request. The problem of resource allocation can be handled by a strongly-entangled quantum network structure* $${{\mathscr {S}}}_{{\mathscr {N}}} $$ *and a random routing* $${{{\mathscr {R}}}}$$ *between the quantum repeaters of* $${{\mathscr {S}}}_{{\mathscr {N}}} $$ *and* $$R_{i} $$.

#### *Proof*

The structure of ${{\mathscr {S}}}_{{\mathscr {N}}}$ allows to $R_{q}^{\left( {{\mathscr {S}}}_{{\mathscr {N}}} \right) }$ to split the $B\left( R_{i}^{\left( {{\mathscr {S}}}_{{\mathscr {N}}} \right) } ,R_{i} \right)$ entanglement throughput request to $\left| {{\mathscr {S}}}_{{\mathscr {N}}} \right|$ smaller, ${B\left( R_{i}^{\left( {{\mathscr {S}}}_{{\mathscr {N}}} \right) } ,R_{i} \right) \Bigg / \left| {{\mathscr {S}}}_{{\mathscr {N}}} \right| }$ requests. As follows, within the structure of ${{\mathscr {S}}}_{{\mathscr {N}}}$, the entangled connection between quantum repeaters $R_{i}^{\left( {{\mathscr {S}}}_{{\mathscr {N}}} \right) }$ and $R_{q}^{\left( {{\mathscr {S}}}_{{\mathscr {N}}} \right) }$, is associated with the following entanglement throughput (Bell states per *C*):

50 $$\begin{aligned} Q\left( R_{i}^{\left( {{\mathscr {S}}}_{{\mathscr {N}}} \right) } ,R_{q}^{\left( {{\mathscr {S}}}_{{\mathscr {N}}} \right) } \right) ={\textstyle \frac{B\left( R_{i}^{\left( {{\mathscr {S}}}_{{\mathscr {N}}} \right) } ,R_{i} \right) }{\left| {{\mathscr {S}}}_{{\mathscr {N}}} \right| }} {\mathrm{,\; for\; }}q=1,\ldots ,\left| {{\mathscr {S}}}_{{\mathscr {N}}} \right| -1,q\ne i. \end{aligned}$$

As follows, using ${{\mathscr {S}}}_{{\mathscr {N}}}$, the entanglement throughputs of all of the $\left| {{\mathscr {S}}}_{{\mathscr {N}}} \right| -1$ entangled connections of $R_{i}^{\left( {{\mathscr {S}}}_{{\mathscr {N}}} \right) }$ are associated with the ${1 \big / \left| {{\mathscr {S}}}_{{\mathscr {N}}} \right| }$-th of the incoming request of $R_{i}^{\left( {{\mathscr {S}}}_{{\mathscr {N}}} \right) }$. Therefore, the incoming of $R_{i}^{\left( {{\mathscr {S}}}_{{\mathscr {N}}} \right) }$ request is divided into $\left| {{\mathscr {S}}}_{{\mathscr {N}}} \right|$ fractions and distributed to the $\left| {{\mathscr {S}}}_{{\mathscr {N}}} \right| -1$ neighbors of $R_{i}^{\left( {{\mathscr {S}}}_{{\mathscr {N}}} \right) }$ in the strongly-entangled structure ${{\mathscr {S}}}_{{\mathscr {N}}}$.

As the quantum repeaters of $${{\mathscr {S}}}_{{\mathscr {N}}} $$ shared the entangled systems with each other, a random routing is utilized from all quantum repeaters of $${{\mathscr {S}}}_{{\mathscr {N}}} $$ to the low-priority node $$R_{i} $$. The request from $$R_{i} $$ to the strongly-entangled structure $${{\mathscr {S}}}_{{\mathscr {N}}} $$ is served via

51 $$\begin{aligned} n_{{\mathscr {P}}} =\left| {{\mathscr {S}}}_{{\mathscr {N}}} \right| \end{aligned}$$

parallel entangled paths $${{\mathscr {P}}}\left( R_{q}^{\left( {{\mathscr {S}}}_{{\mathscr {N}}} \right) } ,R_{i} \right) $$ between the quantum repeaters of $${{\mathscr {S}}}_{{\mathscr {N}}} $$ and $$R_{i} $$.

Therefore, the source of a $${{\mathscr {P}}}\left( R_{q}^{\left( {{\mathscr {S}}}_{{\mathscr {N}}} \right) } ,R_{i} \right) $$ entangled path is the *q*-th quantum repeater $$R_{q}^{\left( {{\mathscr {S}}}_{{\mathscr {N}}} \right) } $$ from $${{\mathscr {S}}}_{{\mathscr {N}}} $$, $$q=1,\ldots ,\left| {{\mathscr {S}}}_{{\mathscr {N}}} \right| $$, while the target is $$R_{i} $$. The $$\left| {{\mathscr {S}}}_{{\mathscr {N}}} \right| $$ parallel entangled paths define the set $${{{\mathscr {R}}}}_{{{\mathscr {S}}}} $$ of random quantum repeaters used in the routing procedure as

52 $$\begin{aligned} {{{\mathscr {R}}}}_{{{\mathscr {S}}}} ={{{\mathscr {R}}}}\left( R_{1}^{\left( {{\mathscr {S}}}_{{\mathscr {N}}} \right) } ,R_{i} \right) \bigcup \ldots \bigcup {{{\mathscr {R}}}}\left( R_{\left| {{\mathscr {S}}}_{{\mathscr {N}}} \right| }^{\left( {{\mathscr {S}}}_{{\mathscr {N}}} \right) } ,R_{i} \right) , \end{aligned}$$

where $${{{\mathscr {R}}}}\left( R_{i}^{\left( {{\mathscr {S}}}_{{\mathscr {N}}} \right) } ,R_{i} \right) $$ identifies a set of random nodes used in the random routing $${{{\mathscr {R}}}}$$ from $$R_{i}^{\left( {{\mathscr {S}}}_{{\mathscr {N}}} \right) } $$ to $$R_{i} $$.

As the entangled paths are established, an $U_{S} \left( R_{q}^{\left( {{\mathscr {S}}}_{{\mathscr {N}}} \right) } \right)$ entanglement swapping operation is applied in all of the $R_{q}^{\left( {{\mathscr {S}}}_{{\mathscr {N}}} \right) }$ quantum repeaters of ${{\mathscr {S}}}_{{\mathscr {N}}}$. The aim of these operations is to swap the entangled connections to the egress quantum repeater $R_{E}^{\left( {{\mathscr {S}}}_{{\mathscr {N}}} \right) }$ of ${{\mathscr {S}}}_{{\mathscr {N}}}$.

The result is $$\left| {{\mathscr {S}}}_{{\mathscr {N}}} \right| $$ entangled connections between $$R_{i} $$ and $$R_{E}^{\left( {{\mathscr {S}}}_{{\mathscr {N}}} \right) } $$, i.e., the set of $$\left| {{\mathscr {S}}}_{{\mathscr {N}}} \right| $$ entangled paths

53 $$\begin{aligned} {{\mathscr {P}}}\left( R_{i} ,B\right) ={{\mathscr {P}}}_{1} \left( R_{i} ,R_{E}^{\left( {{\mathscr {S}}}_{{\mathscr {N}}} \right) } \right) \bigcup \ldots \bigcup {{\mathscr {P}}}_{\left| {{\mathscr {S}}}_{{\mathscr {N}}} \right| } \left( R_{i} ,R_{E}^{\left( {{\mathscr {S}}}_{{\mathscr {N}}} \right) } \right) \end{aligned}$$

such that the $B_{F} \left( {{\mathscr {P}}}\left( R_{i} ,R_{E}^{\left( {{\mathscr {S}}}_{{\mathscr {N}}} \right) } \right) \right)$ entanglement throughput of entangled path ${{\mathscr {P}}}_{j} \left( R_{i} ,R_{E}^{\left( {{\mathscr {S}}}_{{\mathscr {N}}} \right) } \right)$ is as

54 $$\begin{aligned} B_{F} \left( {{\mathscr {P}}}_{j} \left( R_{i} ,R_{E}^{\left( {{\mathscr {S}}}_{{\mathscr {N}}} \right) } \right) \right) =B\left( R_{q}^{\left( {{\mathscr {S}}}_{{\mathscr {N}}} \right) } ,R_{i} \right) ={\textstyle \frac{1}{\left| {{\mathscr {S}}}_{{\mathscr {N}}} \right| }} B\left( R_{i} \right) , \end{aligned}$$

where $$B\left( R_{i} \right) $$ is the total entanglement throughput request of $$R_{i} $$ (Bell states per *C*), since the entangled path of $$R_{q}^{\left( {{\mathscr {S}}}_{{\mathscr {N}}} \right) } $$ and $$R_{i} $$ is swapped to the path between $$R_{E}^{\left( {{\mathscr {S}}}_{{\mathscr {N}}} \right) } $$ and $$R_{i} $$ via a swapping $$U_{S} \left( R_{q}^{\left( {{\mathscr {S}}}_{{\mathscr {N}}} \right) } \right) $$ in $$R_{q}^{\left( {{\mathscr {S}}}_{{\mathscr {N}}} \right) } $$.

Therefore the sum of the entanglement throughput of the $\left| {{\mathscr {S}}}_{{\mathscr {N}}} \right|$ entangled paths (Bell states per *C*) is

55 $$\begin{aligned} \sum _{j=1}^{\left| {{\mathscr {S}}}_{{\mathscr {N}}} \right| }B_{F} \left( {{\mathscr {P}}}_{j} \left( R_{i} ,R_{E}^{\left( {{\mathscr {S}}}_{{\mathscr {N}}} \right) } \right) \right) =\sum _{q=1}^{\left| {{\mathscr {S}}}_{{\mathscr {N}}} \right| }B\left( R_{q}^{\left( {{\mathscr {S}}}_{{\mathscr {N}}} \right) } ,R_{i} \right) =B\left( R_{i} \right) , \end{aligned}$$

thus it equals to the entanglement throughput request received from the low-priority node $$R_{i} $$.

Assuming that there are $$n_{c} $$ low-priority quantum nodes in *N* all with different entanglement throughput requests, the $${{\mathscr {S}}}_{{\mathscr {N}}} $$ strongly-entangled structure has to serve all of these $$n_{c} $$ low-priority quantum nodes simultaneously. In this case, the steps detailed above are established in parallel for all of the $$n_{c} $$ low-priority nodes, thus the $$n_{\Sigma {{\mathscr {P}}}} $$ total number of parallel entangled connections established via the $${{\mathscr {S}}}_{{\mathscr {N}}} $$ structure is

56 $$\begin{aligned} n_{\Sigma {{\mathscr {P}}}} =n_{c} \left| {{\mathscr {S}}}_{{\mathscr {N}}} \right| . \end{aligned}$$

As the ${\mathrm{L}}_{l} \left( R_{i} ,R_{E}^{\left( {{\mathscr {S}}}_{{\mathscr {N}}} \right) } \right)$ entangled connection is built up via the entanglement swapping in $R_{I}^{\left( {{\mathscr {S}}}_{{\mathscr {N}}} \right) }$, an arbitrary routing from $R_{E}^{\left( {{\mathscr {S}}}_{{\mathscr {N}}} \right) }$ to $D\left( R_{i} \right)$ can be used to construct the entangled connection ${\mathrm{L}}_{l} \left( R_{E}^{\left( {{\mathscr {S}}}_{{\mathscr {N}}} \right) } ,D\left( R_{i} \right) \right)$. Then, an entanglement swapping in $R_{E}^{\left( {{\mathscr {S}}}_{{\mathscr {N}}} \right) }$ yields the long-distance ${\mathrm{L}}_{l} \left( R_{i} ,D\left( R_{i} \right) \right)$ entangled connection.

$$\square $$

The construction method of a strongly-entangled structure is given Procedure 2.

**Figure 3:**
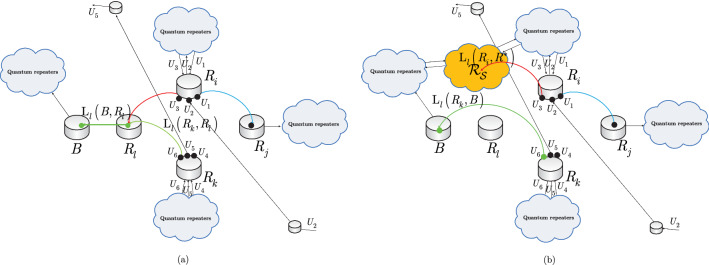
Entanglement distribution with resource balancing in the quantum Internet. (**a**) Low-priority quantum repeaters. Users $$U_{3} $$ and $$U_{6} $$ would like to share an entangled connection with *B* through $$R_{l} $$. Quantum repeater $$R_{l} $$ has only a single density matrix from *B* available for the entanglement swapping via entangled connection $$\mathrm{L}_{l} \left( R_{k} ,B\right) $$, and as a corollary, $$R_{l} $$ can serve only $$U_{3} $$ or $$U_{6} $$. Users $$U_{1} $$, $$U_{2} $$, and $$U_{5} $$ are served directly, since these users have no common resource requirements. The quantum repeater node $$R_{l} $$ serves $$U_{6} $$, thus establishing the entangled connection $${\mathrm{L}}_{l} \left( R_{k} ,R_{l} \right) $$ between $$R_{k} $$ and $$R_{l} $$. (**b**) Resource balancing via random routing. User $$U_{6} $$ establishes the distant entangled connection $${\mathrm{L}}_{l} \left( R_{k} ,B\right) $$ with *B* through $$R_{l} $$ (depicted by the green line). For a seamless transition of resource saving, a random quantum repeater is selected for user $$U_{3} $$ from the set $${{{\mathscr {R}}}}_{{{\mathscr {S}}}} $$ of random quantum repeaters ($${{{\mathscr {R}}}}_{{{\mathscr {S}}}} $$ is realized by the strongly-entangled structure $${{\mathscr {S}}}_{{\mathscr {N}}} $$) to establish the entangled connection $${\mathrm{L}}_{l} \left( R_{i} ,R^{*} \right) $$ (depicted by the red line), where $$R^{*} \in {{{\mathscr {R}}}}_{{{\mathscr {S}}}} $$ is a quantum repeater from $${{{\mathscr {R}}}}_{{{\mathscr {S}}}} $$.

Figure 3 depicts a quantum Internet scenario with the requirement of resource balancing in the quantum repeaters of the entanglement distribution process.

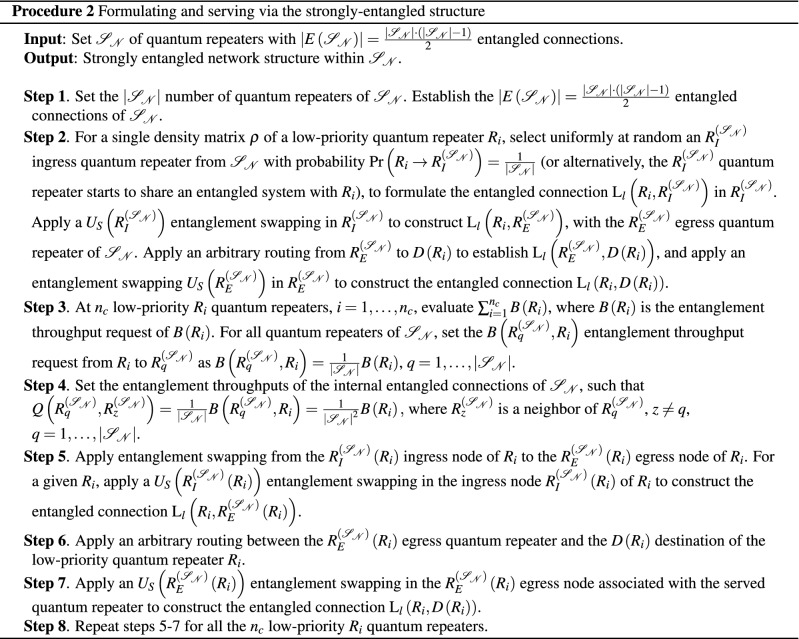



### Resource balancing

#### Theorem 3

(Capability of the strongly-entangled structure). *The strongly-entangled structure* $${{\mathscr {S}}}_{{\mathscr {N}}} $$ *provides a structure to serve all the* $$n_{c} $$ *low-priority quantum nodes simultaneously.*

#### *Proof*

Using the metrics defined in “Capability of a strongly-entangled structure” section, first we derive some relevant attributes of ${{\mathscr {S}}}_{{\mathscr {N}}}$.

From (14), the $${{{\mathscr {F}}}}\left( R_{q}^{\left( {{\mathscr {S}}}_{{\mathscr {N}}} \right) } \right) $$ fanout (ratio of the $$W\left( R_{q}^{\left( {{\mathscr {S}}}_{{\mathscr {N}}} \right) } \right) $$ total entanglement throughput (14) within $${{\mathscr {S}}}_{{\mathscr {N}}} $$ and the $$B\left( R_{q}^{\left( {{\mathscr {S}}}_{{\mathscr {N}}} \right) } ,{{\mathscr {S}}}_{n_{c} } \right) $$ incoming request from the low-priority quantum repeaters, (12) of a quantum repeater $$R_{q}^{\left( {{\mathscr {S}}}_{{\mathscr {N}}} \right) } $$ at $$n_{c} $$ low-priority quantum repeaters is defined as

57 $$\begin{aligned} \begin{aligned} \mathscr {F}\left( R_{q}^{\left( {{\mathscr {S}}_{\mathscr {N}}} \right) } \right)&:=\tfrac{W\left( R_{q}^{\left( {{\mathscr {S}}_{\mathscr {N}}} \right) } \right) }{B\left( R_{q}^{\left( {{\mathscr {S}}_{\mathscr {N}}} \right) },{{\mathscr {S}}_{{{n}_{c}}}} \right) } \\&=\tfrac{\left( \left| {{\mathscr {S}}_{\mathscr {N}}} \right| -1 \right) \tfrac{1}{{{\left| {{\mathscr {S}}_{\mathscr {N}}} \right| }^{2}}}\left( \sum \limits _{i=1}^{{{n}_{c}}}{B\left( {{R}_{i}} \right) } \right) }{\tfrac{1}{\left| {{\mathscr {S}}_{\mathscr {N}}} \right| }\left( \sum \limits _{i=1}^{{{n}_{c}}}{B\left( {{R}_{i}} \right) } \right) } \\&=\tfrac{1}{\left| {{\mathscr {S}}_{\mathscr {N}}} \right| }\left( \left| {{\mathscr {S}}_{\mathscr {N}}} \right| -1 \right) \\&=\left( 1-\tfrac{1}{\left| {{\mathscr {S}}_{\mathscr {N}}} \right| } \right) , \end{aligned} \end{aligned}$$

and the ${{{\mathscr {F}}}}\left( {{\mathscr {S}}}_{{\mathscr {N}}} \right)$ fanout of ${{\mathscr {S}}}_{{\mathscr {N}}}$ as the maximum fanout among the quantum repeaters of ${{\mathscr {S}}}_{{\mathscr {N}}}$ as

58 $$\begin{aligned} {{{\mathscr {F}}}}\left( {{\mathscr {S}}}_{{\mathscr {N}}} \right) :=\mathop {\max }\limits _{q} {{{\mathscr {F}}}}\left( R_{q}^{\left( {{\mathscr {S}}}_{{\mathscr {N}}} \right) } \right) =\left( 1-{\textstyle \frac{1}{\left| {{\mathscr {S}}}_{{\mathscr {N}}} \right| }} \right) , \end{aligned}$$

such that

59 $$\begin{aligned} \mathop {\max }\limits _{q} {\textstyle \frac{W\left( R_{q}^{\left( {{\mathscr {S}}}_{{\mathscr {N}}} \right) } \right) }{{\textstyle \frac{1}{\left| {{\mathscr {S}}}_{{\mathscr {N}}} \right| ^{2} }} \left( \sum _{i=1}^{n_{c} }B\left( R_{i} \right) \right) }} \ge {\textstyle \frac{Z\left( {{\mathscr {S}}}_{{\mathscr {N}}} \right) }{B\left( {{\mathscr {S}}}_{{\mathscr {N}}} \right) }} , \end{aligned}$$

by theory^135,136^, where $$Z\left( {{\mathscr {S}}}_{{\mathscr {N}}} \right) $$ is as given in (15), while $$B\left( {{\mathscr {S}}}_{{\mathscr {N}}} \right) $$ is the total requests from the $$n_{c} $$ quantum repeaters to $${{\mathscr {S}}}_{{\mathscr {N}}} $$ (Bell states per *C*) as

60 $$\begin{aligned} B\left( {{\mathscr {S}}}_{{\mathscr {N}}} \right) =\sum _{q=1}^{\left| {{\mathscr {S}}}_{{\mathscr {N}}} \right| }B\left( R_{q}^{\left( {{\mathscr {S}}}_{{\mathscr {N}}} \right) } ,{{\mathscr {S}}}_{n_{c} } \right) =\sum _{i=1}^{n_{c} }B\left( R_{i} \right) . \end{aligned}$$

Thus, (59) can be rewritten as

61 $$\begin{aligned} {{{\mathscr {F}}}}\left( {{\mathscr {S}}}_{{\mathscr {N}}} \right) ={\textstyle \frac{\left( \left| {{\mathscr {S}}}_{{\mathscr {N}}} \right| -1\right) {\textstyle \frac{1}{\left| {{\mathscr {S}}}_{{\mathscr {N}}} \right| }} \left( \sum _{i=1}^{n_{c} }B\left( R_{i} \right) \right) }{\left| {{\mathscr {S}}}_{{\mathscr {N}}} \right| \left( {\textstyle \frac{1}{\left| {{\mathscr {S}}}_{{\mathscr {N}}} \right| }} \left( \sum _{i=1}^{n_{c} }B\left( R_{i} \right) \right) \right) }} =\left( 1-{\textstyle \frac{1}{\left| {{\mathscr {S}}}_{{\mathscr {N}}} \right| }} \right) . \end{aligned}$$

As a corollary, ${{{\mathscr {F}}}}\left( {{\mathscr {S}}}_{{\mathscr {N}}} \right) \le 1$ for any $\left| {{\mathscr {S}}}_{{\mathscr {N}}} \right| \ge 1$, while in a classical full-mesh structure ${{{\mathscr {M}}}}$, the fanout is lower bounded by 1, i.e. ${{{\mathscr {F}}}}\left( {{{\mathscr {M}}}}\right) \ge 1$. As follows, the ${{{\mathscr {F}}}}\left( {{\mathscr {S}}}_{{\mathscr {N}}} \right) \le 1$ property is strictly resulted from the attributes of the quantum structure (such as entanglement swapping), and it cannot be achieved within any classical full-mesh structure-based uniform load-balancing^135,136^.

Note, that in (61) it is assumed that within the structure of ${{\mathscr {S}}}_{{\mathscr {N}}}$, all the $R_{q}^{\left( {{\mathscr {S}}}_{{\mathscr {N}}} \right) }$ are associated with the same $B\left( R_{q}^{\left( {{\mathscr {S}}}_{{\mathscr {N}}} \right) } ,{{\mathscr {S}}}_{n_{c} } \right)$ values (see (12)), and a corollary, the throughputs of the entangled connections within ${{\mathscr {S}}}_{{\mathscr {N}}}$ are set equally to $Q\left( R_{q}^{\left( {{\mathscr {S}}}_{{\mathscr {N}}} \right) } ,R_{z}^{\left( {{\mathscr {S}}}_{{\mathscr {N}}} \right) } \right)$ (see (13)), since each quantum repeater receive the same amount of incoming request. Let us to derive ${{{\mathscr {F}}}}\left( {{\mathscr {S}}}_{{\mathscr {N}}} \right)$ for the case if the $B\left( R_{q}^{\left( {{\mathscr {S}}}_{{\mathscr {N}}} \right) } ,{{\mathscr {S}}}_{n_{c} } \right)$ values of ${{\mathscr {S}}}_{{\mathscr {N}}}$ are not equally set, while the condition

62 $$\begin{aligned} \sum _{q=1}^{\left| {{\mathscr {S}}}_{{\mathscr {N}}} \right| }B\left( R_{q}^{\left( {{\mathscr {S}}}_{{\mathscr {N}}} \right) } ,{{\mathscr {S}}}_{n_{c} } \right) =\sum _{i=1}^{n_{c} }B\left( R_{i} \right) . \end{aligned}$$

holds for the $B\left( R_{q}^{\left( {{\mathscr {S}}}_{{\mathscr {N}}} \right) } ,{{\mathscr {S}}}_{n_{c} } \right)$ values in ingress quantum repeaters.

In this case, (15) is as

63 $$\begin{aligned} Z\left( {{\mathscr {S}}}_{{\mathscr {N}}} \right) =\sum _{q=1}^{\left| {{\mathscr {S}}}_{{\mathscr {N}}} \right| }\left( \left| {{\mathscr {S}}}_{{\mathscr {N}}} \right| -1\right) {\textstyle \frac{1}{\left| {{\mathscr {S}}}_{{\mathscr {N}}} \right| }} B\left( R_{q}^{\left( {{\mathscr {S}}}_{{\mathscr {N}}} \right) } ,{{\mathscr {S}}}_{n_{c} } \right) , \end{aligned}$$

thus ${{{\mathscr {F}}}}\left( {{\mathscr {S}}}_{{\mathscr {N}}} \right)$ is yielded as

64 $$\begin{aligned} \begin{aligned} \mathscr {F}\left( {{\mathscr {S}}_{\mathscr {N}}} \right)&=\tfrac{\sum \limits _{q=1}^{\left| {{\mathscr {S}}_{\mathscr {N}}} \right| }{\left( \left| {{\mathscr {S}}_{\mathscr {N}}} \right| -1 \right) \tfrac{1}{\left| {{\mathscr {S}}_{\mathscr {N}}} \right| }B\left( R_{q}^{\left( {{\mathscr {S}}_{\mathscr {N}}} \right) },{{\mathscr {S}}_{{{n}_{c}}}} \right) }}{B\left( {{\mathscr {S}}_{\mathscr {N}}} \right) } \\&=\tfrac{\left( \left( \left| {{\mathscr {S}}_{\mathscr {N}}} \right| -1 \right) \tfrac{1}{\left| {{\mathscr {S}}_{\mathscr {N}}} \right| } \right) \sum \limits _{i=1}^{{{n}_{c}}}{B\left( {{R}_{i}} \right) }}{\sum \limits _{i=1}^{{{n}_{c}}}{B\left( {{R}_{i}} \right) }} \\&=\left( \left| {{\mathscr {S}}_{\mathscr {N}}} \right| -1 \right) \tfrac{1}{\left| {{\mathscr {S}}_{\mathscr {N}}} \right| }, \end{aligned} \end{aligned}$$

thus (64) picks up its minimum (61) if the incoming density matrices of ${{\mathscr {S}}}_{{\mathscr {N}}}$ are not uniformly distributed.

On the other hand, if

65 $$\begin{aligned} \sum _{q=1}^{\left| {{\mathscr {S}}}_{{\mathscr {N}}} \right| }B\left( R_{q}^{\left( {{\mathscr {S}}}_{{\mathscr {N}}} \right) } ,{{\mathscr {S}}}_{n_{c} } \right) <\sum _{i=1}^{n_{c} }B\left( R_{i} \right) , \end{aligned}$$

such that

66 $$\begin{aligned} \sum _{q=1}^{\left| {{\mathscr {S}}}_{{\mathscr {N}}} \right| }B\left( R_{q}^{\left( {{\mathscr {S}}}_{{\mathscr {N}}} \right) } ,{{\mathscr {S}}}_{n_{c} } \right) +x=\sum _{i=1}^{n_{c} }B\left( R_{i} \right) \end{aligned}$$

while the internal entangled connections of ${{\mathscr {S}}}_{{\mathscr {N}}}$ are set with relation $\sum _{q}Q\left( R_{q}^{\left( {{\mathscr {S}}}_{{\mathscr {N}}} \right) } ,R_{z}^{\left( {{\mathscr {S}}}_{{\mathscr {N}}} \right) } \right) =\sum _{i=1}^{n_{c} }B\left( R_{i} \right)$, then

67 $$\begin{aligned} \begin{aligned} \mathscr {F}\left( {{\mathscr {S}}_{\mathscr {N}}} \right)&=\tfrac{\left( \left( \left| {{\mathscr {S}}_{\mathscr {N}}} \right| -1 \right) \tfrac{1}{\left| {{\mathscr {S}}_{\mathscr {N}}} \right| } \right) \sum \limits _{i=1}^{{{n}_{c}}}{B\left( {{R}_{i}} \right) }}{B\left( {{\mathscr {S}}_{\mathscr {N}}} \right) -x} \\&>\left( \left| {{\mathscr {S}}_{\mathscr {N}}} \right| -1 \right) \tfrac{1}{\left| {{\mathscr {S}}_{\mathscr {N}}} \right| }. \end{aligned} \end{aligned}$$

On the relation of the incoming request and the internal entanglement throughputs of the entangled connections some derivations are as follows.

Let $$B\left( R_{q}^{\left( {{\mathscr {S}}}_{{\mathscr {N}}} \right) } ,{{\mathscr {S}}}_{n_{c} } \right) $$ be the entanglement throughput request from the $$n_{c} $$ low-priority nodes to $$R_{q}^{\left( {{\mathscr {S}}}_{{\mathscr {N}}} \right) } $$ (Bell states per *C*), and let $$R_{E}^{\left( {{\mathscr {S}}}_{{\mathscr {N}}} \right) } $$ be the egress node of the requests with entangled connection $${\mathrm{L}}_{l} \left( R_{q}^{\left( {{\mathscr {S}}}_{{\mathscr {N}}} \right) } ,R_{E}^{\left( {{\mathscr {S}}}_{{\mathscr {N}}} \right) } \right) $$.

If the entanglement throughput $Q\left( R_{q}^{\left( {{\mathscr {S}}}_{{\mathscr {N}}} \right) } ,R_{E}^{\left( {{\mathscr {S}}}_{{\mathscr {N}}} \right) } \right)$ within ${{\mathscr {S}}}_{{\mathscr {N}}}$ is set as

68 $$\begin{aligned} Q\left( R_{q}^{\left( {{\mathscr {S}}}_{{\mathscr {N}}} \right) } ,R_{E}^{\left( {{\mathscr {S}}}_{{\mathscr {N}}} \right) } \right) \ge B\left( R_{q}^{\left( {{\mathscr {S}}}_{{\mathscr {N}}} \right) } ,{{\mathscr {S}}}_{n_{c} } \right) , \end{aligned}$$

then a request from $R_{q}^{\left( {{\mathscr {S}}}_{{\mathscr {N}}} \right) }$ to $R_{E}^{\left( {{\mathscr {S}}}_{{\mathscr {N}}} \right) }$ can be served, while if

69 $$\begin{aligned} Q\left( R_{q}^{\left( {{\mathscr {S}}}_{{\mathscr {N}}} \right) } ,R_{E}^{\left( {{\mathscr {S}}}_{{\mathscr {N}}} \right) } \right) <B\left( R_{q}^{\left( {{\mathscr {S}}}_{{\mathscr {N}}} \right) } ,{{\mathscr {S}}}_{n_{c} } \right) , \end{aligned}$$

the request $B\left( R_{q}^{\left( {{\mathscr {S}}}_{{\mathscr {N}}} \right) } ,{{\mathscr {S}}}_{n_{c} } \right)$ is served through different ${\mathrm{L}}_{l} \left( R_{i}^{\left( {{\mathscr {S}}}_{{\mathscr {N}}} \right) } ,R_{j}^{\left( {{\mathscr {S}}}_{{\mathscr {N}}} \right) } \right)$ entangled connections in ${{\mathscr {S}}}_{{\mathscr {N}}}$, such that

70 $$\begin{aligned} \sum _{i<j}Q\left( R_{i}^{\left( {{\mathscr {S}}}_{{\mathscr {N}}} \right) } ,R_{j}^{\left( {{\mathscr {S}}}_{{\mathscr {N}}} \right) } \right) \ge Q\left( R_{q}^{\left( {{\mathscr {S}}}_{{\mathscr {N}}} \right) } ,R_{E}^{\left( {{\mathscr {S}}}_{{\mathscr {N}}} \right) } \right) , \end{aligned}$$

and

71 $$\begin{aligned} \sum _{i<j}Q\left( R_{i}^{\left( {{\mathscr {S}}}_{{\mathscr {N}}} \right) } ,R_{j}^{\left( {{\mathscr {S}}}_{{\mathscr {N}}} \right) } \right) \ge B\left( R_{q}^{\left( {{\mathscr {S}}}_{{\mathscr {N}}} \right) } ,{{\mathscr {S}}}_{n_{c} } \right) . \end{aligned}$$

Assuming that (68) holds for all quantum repeaters of ${{\mathscr {S}}}_{{\mathscr {N}}}$, then

72 $$\begin{aligned} \sum _{q\ne E}Q\left( R_{q}^{\left( {{\mathscr {S}}}_{{\mathscr {N}}} \right) } ,R_{E}^{\left( {{\mathscr {S}}}_{{\mathscr {N}}} \right) } \right) \ge \sum _{q\ne E}B\left( R_{q}^{\left( {{\mathscr {S}}}_{{\mathscr {N}}} \right) } ,{{\mathscr {S}}}_{n_{c} } \right) , \end{aligned}$$

while if $q=E$, then the $R_{q}^{\left( {{\mathscr {S}}}_{{\mathscr {N}}} \right) }$ node is also the egress node, thus the aim is to achieve an arbitrary routing from $R_{q}^{\left( {{\mathscr {S}}}_{{\mathscr {N}}} \right) }$ to the distant node associated with the incoming request that is not part of the structure ${{\mathscr {S}}}_{{\mathscr {N}}}$.

The proof is concluded here. $\square$

**Figure 4:**
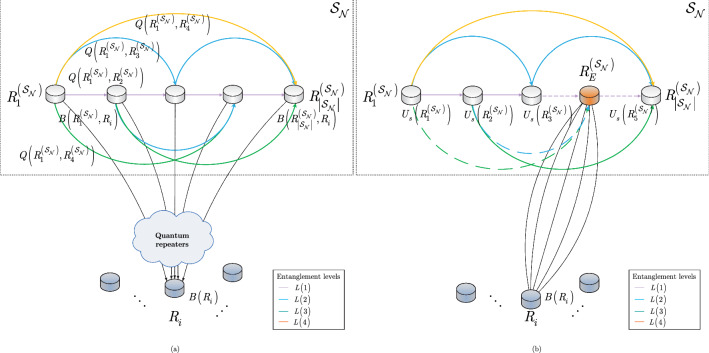
The strongly-entangled structure $${{\mathscr {S}}}_{{\mathscr {N}}} $$ as formed by $$\left| {{\mathscr {S}}}_{{\mathscr {N}}} \right| $$ quantum repeaters and $$\left| E\left( {{\mathscr {S}}}_{{\mathscr {N}}} \right) \right| $$ entangled connections with heterogeneous entanglement levels, where $$\left| {{\mathscr {S}}}_{{\mathscr {N}}} \right| =5$$, $$\left| E\left( {{\mathscr {S}}}_{{\mathscr {N}}} \right) \right| ={\textstyle \frac{\left| {{\mathscr {S}}}_{{\mathscr {N}}} \right| \cdot \left( \left| {{\mathscr {S}}}_{{\mathscr {N}}} \right| -1\right) }{2}} $$ , and $${{{\mathscr {F}}}}\left( {{\mathscr {S}}}_{{\mathscr {N}}} \right) =\left( 1-{\textstyle \frac{1}{\left| {{\mathscr {S}}}_{{\mathscr {N}}} \right| }} \right) $$. (**a**) The low-priority node $$R_{i} $$ is associated with the entanglement throughput request $$B\left( R_{i} \right) $$. The $$\left| {{\mathscr {S}}}_{{\mathscr {N}}} \right| $$ quantum repeaters of $${{\mathscr {S}}}_{{\mathscr {N}}} $$ establish $$\left| {{\mathscr {S}}}_{{\mathscr {N}}} \right| $$ entangled connections with $$R_{i} $$ (depicted by the outgoing dashed black lines), with each connection having entanglement throughput $$B\left( R_{q}^{\left( {{\mathscr {S}}}_{{\mathscr {N}}} \right) } ,R_{i} \right) ={\textstyle \frac{1}{\left| {{\mathscr {S}}}_{{\mathscr {N}}} \right| }} B\left( R_{i} \right) $$, where $$\sum _{q=1}^{\left| {{\mathscr {S}}}_{{\mathscr {N}}} \right| }B\left( R_{q}^{\left( {{\mathscr {S}}}_{{\mathscr {N}}} \right) } ,R_{i} \right) =B\left( R_{i} \right) $$. A given quantum repeater $$R_{q}^{\left( {{\mathscr {S}}}_{{\mathscr {N}}} \right) } $$ of $${{\mathscr {S}}}_{{\mathscr {N}}} $$ establishes $$\left| {{\mathscr {S}}}_{{\mathscr {N}}} \right| -1$$ entangled connections within $${{\mathscr {S}}}_{{\mathscr {N}}} $$, each with entanglement throughput $$Q\left( R_{q}^{\left( {{\mathscr {S}}}_{{\mathscr {N}}} \right) } ,R_{z}^{\left( {{\mathscr {S}}}_{{\mathscr {N}}} \right) } \right) ={\textstyle \frac{1}{\left| {{\mathscr {S}}}_{{\mathscr {N}}} \right| }} B\left( R_{q}^{\left( {{\mathscr {S}}}_{{\mathscr {N}}} \right) } ,R_{i} \right) $$, where $$R_{z}^{\left( {{\mathscr {S}}}_{{\mathscr {N}}} \right) } $$ is a neighbor of $$R_{q}^{\left( {{\mathscr {S}}}_{{\mathscr {N}}} \right) } $$. (**b**) Each of the $$\left| {{\mathscr {S}}}_{{\mathscr {N}}} \right| -1$$ quantum repeaters of $${{\mathscr {S}}}_{{\mathscr {N}}} $$ applies entanglement swapping $$U_{S} $$ to establish the entangled connection between $$R_{i} $$ and the egress quantum repeater $$R_{E}^{\left( {{\mathscr {S}}}_{{\mathscr {N}}} \right) } $$ of $${{\mathscr {S}}}_{{\mathscr {N}}} $$. Then, an arbitrary routing is applied to establish the entangled connection between $$R_{i} $$ and the destination node $$D\left( R_{i} \right) $$ of $$R_{i} $$. The request from $$R_{i} $$ to the strongly-entangled structure $${{\mathscr {S}}}_{{\mathscr {N}}} $$ is served via $$n_{{\mathscr {P}}} =\left| {{\mathscr {S}}}_{{\mathscr {N}}} \right| $$ parallel entangled paths $${{\mathscr {P}}}\left( R_{q}^{\left( {{\mathscr {S}}}_{{\mathscr {N}}} \right) } ,R_{i} \right) $$ between the quantum repeaters of $${{\mathscr {S}}}_{{\mathscr {N}}} $$ and $$R_{i} $$. The dashed entangled connections are rebuilt within $${{\mathscr {S}}}_{{\mathscr {N}}} $$ after the entanglement swapping operations.

The schematic model of the strongly-entangled structure ${{\mathscr {S}}}_{{\mathscr {N}}}$ is illustrated in Fig. 4.

#### Lemma 2

(Resource-balancing efficiency of the strongly-entangled structure $${{\mathscr {S}}}_{{\mathscr {N}}} $$ for $$n_{c} $$ low-priority nodes).*In terms of fanout minimization and total traffic minimization, the strongly-entangled quantum network structure* $${{\mathscr {S}}}_{{\mathscr {N}}} $$ *is two times more efficient than a classical full-mesh network structure* $${{{\mathscr {M}}}}$$.

#### *Proof*

First, we compare the fanout coefficients of the classical full-mesh structure ${{{\mathscr {M}}}}$ and the strongly-entangled quantum network ${{\mathscr {S}}}_{{\mathscr {N}}}$. Then, we compare the total amount of traffic within the structures of ${{{\mathscr {M}}}}$ and ${{\mathscr {S}}}_{{\mathscr {N}}}$.

For simplicity, let us assume that $\left| {{{\mathscr {M}}}}\right| =\left| {{\mathscr {S}}}_{{\mathscr {N}}} \right|$ and that the nodes of the structures are associated with the same incoming traffic (measured in the number of packets for ${{{\mathscr {M}}}}$, and the number of density matrices for ${{\mathscr {S}}}_{{\mathscr {N}}}$):

73 $$\begin{aligned} T\left( x_{q}^{\left( {{{\mathscr {M}}}}\right) } ,x_{i} \right) =B\left( R_{q}^{\left( {{\mathscr {S}}}_{{\mathscr {N}}} \right) } ,R_{i} \right) , \end{aligned}$$

where $$T\left( \cdot \right) $$ is the traffic of $${{{\mathscr {M}}}}$$, $$B\left( \cdot \right) $$ is the traffic of $${{\mathscr {S}}}_{{\mathscr {N}}} $$, $$x_{i} $$ is a source node, $$x_{q}^{\left( {{{\mathscr {M}}}}\right) } $$ is the *q*-th node of $${{{\mathscr {M}}}}$$, $$R_{i} $$ is a source quantum repeater, and $$R_{q}^{\left( {{\mathscr {S}}}_{{\mathscr {N}}} \right) } $$ is the *q*-th quantum repeater of $${{\mathscr {S}}}_{{\mathscr {N}}} $$.

It can be verified^135,136^, that for structure ${{{\mathscr {M}}}}$, the fanout coefficient ${{{\mathscr {F}}}}\left( {{{\mathscr {M}}}}\right)$ is

74 $$\begin{aligned} {{{\mathscr {F}}}}\left( {{{\mathscr {M}}}}\right) =2\left( \left| {{{\mathscr {M}}}}\right| -1\right) {\textstyle \frac{1}{\left| {{{\mathscr {M}}}}\right| }} =2\left( 1-{\textstyle \frac{1}{\left| {{{\mathscr {M}}}}\right| }} \right) , \end{aligned}$$

where ${{{\mathscr {F}}}}\left( {{\mathscr {S}}}_{{\mathscr {N}}} \right)$ is as in (58). The fanout of the entangled structure is half of the fanout of ${{{\mathscr {M}}}}$; thus, $\mu \left( {{{\mathscr {F}}}}\left( {{\mathscr {S}}}_{{\mathscr {N}}} \right) ,{{{\mathscr {F}}}}\left( {{{\mathscr {M}}}}\right) \right)$, the ratio of ${{{\mathscr {F}}}}\left( {{\mathscr {S}}}_{{\mathscr {N}}} \right)$ to ${{{\mathscr {F}}}}\left( {{{\mathscr {M}}}}\right)$, trivially follows: $\mu \left( {{{\mathscr {F}}}}\left( {{\mathscr {S}}}_{{\mathscr {N}}} \right) ,{{{\mathscr {F}}}}\left( {{{\mathscr {M}}}}\right) \right) ={\textstyle \frac{{{{\mathscr {F}}}}\left( {{\mathscr {S}}}_{{\mathscr {N}}} \right) }{{{{\mathscr {F}}}}\left( {{{\mathscr {M}}}}\right) }} ={\textstyle \frac{1}{2}}$.

Therefore, in terms of fanout minimization, the strongly-entangled structure is two times more efficient than a classical full-mesh structure.

In terms of the total traffic required within the structures, the results are as follows.

It can be proven that in the classical full-mesh structure $${{{\mathscr {M}}}}$$, two phases of communications are required to establish a communication between a low-priority node $$x_{i} $$ and an egress node $$x_{E}^{\left( {{{\mathscr {M}}}}\right) } $$ of $${{{\mathscr {M}}}}$$. In the first phase, the ingress node $$x_{I}^{\left( {{{\mathscr {M}}}}\right) } $$ of $${{{\mathscr {M}}}}$$ transmits the incoming packet to a random intermediate node of $${{{\mathscr {M}}}}$$. In the second phase, the packet is transmitted from $$x_{z}^{\left( {{{\mathscr {M}}}}\right) } $$ to the exit node $$x_{E}^{\left( {{{\mathscr {M}}}}\right) } $$ of $${{{\mathscr {M}}}}$$. Accordingly, an incoming packet traverses $${{{\mathscr {M}}}}$$ twice^135,136^.

On the other hand, in the strongly-entangled structure ${{\mathscr {S}}}_{{\mathscr {N}}}$, only the first phase is required for seamless routing. The second step can be replaced via the entanglement swapping operator; thus, the incoming densities can be entangled with the target node without a second phase transmission.

In $${{\mathscr {S}}}_{{\mathscr {N}}} $$, all quantum repeaters share an entangled connection with the low-priority node; thus, in a quantum repeater $$R_{q}^{\left( {{\mathscr {S}}}_{{\mathscr {N}}} \right) } $$ of $${{\mathscr {S}}}_{{\mathscr {N}}} $$ only an entanglement swapping $$U_{S} \left( R_{q}^{\left( {{\mathscr {S}}}_{{\mathscr {N}}} \right) } \right) $$ is required to establish an entangled connection between the low-priority node $$R_{i} $$ and the egress quantum repeater $$R_{E}^{\left( {{\mathscr {S}}}_{{\mathscr {N}}} \right) } $$ of $${{\mathscr {S}}}_{{\mathscr {N}}} $$. Therefore, as the entangled path is established from $$R_{E}^{\left( {{\mathscr {S}}}_{{\mathscr {N}}} \right) } $$ to $$D\left( R_{i} \right) $$, a swapping $$U_{S} \left( R_{E}^{\left( {{\mathscr {S}}}_{{\mathscr {N}}} \right) } \right) $$ in $$R_{E}^{\left( {{\mathscr {S}}}_{{\mathscr {N}}} \right) } $$ connects $$D\left( R_{i} \right) $$ with the low-priority node $$R_{i} $$. Accordingly, in the strongly-entangled structure $${{\mathscr {S}}}_{{\mathscr {N}}} $$, it is enough to apply only one phase to serve $$R_{i} $$ via $$R_{E}^{\left( {{\mathscr {S}}}_{{\mathscr {N}}} \right) } $$, whereas $${{{\mathscr {M}}}}$$ requires two phases.

The corollaries for the amount of traffic within the structures are as follows. In ${{{\mathscr {M}}}}$, each node uniformly load-balances its incoming traffic to the other nodes of the structure, regardless of the destination, and then all packets are delivered to the final destination via an egress node by an arbitrary routing^135,136^. The two phases within ${{{\mathscr {M}}}}$ require a total traffic

75 $$\begin{aligned} T\left( x_{i} ,{{{\mathscr {M}}}}\right) ={\textstyle \frac{2\left( \left| {{{\mathscr {M}}}}\right| -1\right) T\left( x_{I}^{\left( {{{\mathscr {M}}}}\right) } ,x_{i} \right) }{\left| {{{\mathscr {M}}}}\right| }} . \end{aligned}$$

In ${{\mathscr {S}}}_{{\mathscr {N}}}$, since only the first phase is required, it reduces the total traffic to

76 $$\begin{aligned} B\left( R_{i} ,{{\mathscr {S}}}_{{\mathscr {N}}} \right) ={\textstyle \frac{\left( \left| {{\mathscr {S}}}_{{\mathscr {N}}} \right| -1\right) B\left( R_{q}^{\left( {{\mathscr {S}}}_{{\mathscr {N}}} \right) } ,R_{i} \right) }{\left| {{\mathscr {S}}}_{{\mathscr {N}}} \right| }} ; \end{aligned}$$

thus from (75) and (76), the ratio of the total transmissions within ${{{\mathscr {M}}}}$ and ${{\mathscr {S}}}_{{\mathscr {N}}}$ is

77 $$\begin{aligned} \mu \left( R_{i} ,x_{i} \right) ={\textstyle \frac{B\left( R_{i} ,{{\mathscr {S}}}_{{\mathscr {N}}} \right) }{T\left( x_{i} ,{{{\mathscr {M}}}}\right) }} ={\textstyle \frac{1}{2}} . \end{aligned}$$

Then, let us further assume that there are $$n_{c} $$ low-priority nodes with a node set $${{\mathscr {S}}}_{n_{c} } $$ and that each node $$x_{i}^{\left( {{{\mathscr {M}}}}\right) } $$ of $${{{\mathscr {M}}}}$$ is an ingress node receiving incoming traffic $$T\left( x_{I,i}^{\left( {{{\mathscr {M}}}}\right) } ,S_{i}^{n_{c} } \right) $$ from $$S_{i}^{n_{c} } $$, where $$S_{i}^{n_{c} } $$ is the *i*-th subset of $${{\mathscr {S}}}_{n_{c} } $$.

In this case, the total traffic in ${{{\mathscr {M}}}}$ is $T\left( {{\mathscr {S}}}_{n_{c} } ,{{{\mathscr {M}}}}\right) =\sum _{i=1}^{\left| {{{\mathscr {M}}}}\right| }{\textstyle \frac{2\left( \left| {{{\mathscr {M}}}}\right| -1\right) T\left( x_{I,i}^{\left( {{{\mathscr {M}}}}\right) } ,S_{i}^{n_{c} } \right) }{\left| {{{\mathscr {M}}}}\right| }}$,

whereas for the structure ${{\mathscr {S}}}_{{\mathscr {N}}}$,

78 $$\begin{aligned} B\left( {{\mathscr {S}}}_{n_{c} } ,{{\mathscr {S}}}_{{\mathscr {N}}} \right) =\sum _{q=1}^{\left| {{\mathscr {S}}}_{{\mathscr {N}}} \right| }{\textstyle \frac{\left( \left| {{\mathscr {S}}}_{{\mathscr {N}}} \right| -1\right) B\left( R_{q}^{\left( {{\mathscr {S}}}_{{\mathscr {N}}} \right) } ,S_{i}^{n_{c} } \right) }{\left| {{\mathscr {S}}}_{{\mathscr {N}}} \right| }} ; \end{aligned}$$

thus, the ratio of the total traffic in the structures is also ${1 \big / 2}$, since

79 $$\begin{aligned} \mu \left( {{\mathscr {S}}}_{{\mathscr {N}}} ,{{{\mathscr {M}}}}\right) ={\textstyle \frac{B\left( {{\mathscr {S}}}_{n_{c} } ,{{\mathscr {S}}}_{{\mathscr {N}}} \right) }{T\left( {{\mathscr {S}}}_{n_{c} } ,{{{\mathscr {M}}}}\right) }} ={\textstyle \frac{1}{2}} . \end{aligned}$$

Assuming that the incoming traffic is the same for all ingress nodes in the structures of ${{{\mathscr {M}}}}$ and ${{\mathscr {S}}}_{{\mathscr {N}}}$, the result in (5.2) simplifies as

80 $$\begin{aligned} T\left( {{\mathscr {S}}}_{n_{c} } ,{{{\mathscr {M}}}}\right) ={\textstyle \frac{\left| {{{\mathscr {M}}}}\right| \left( \left| {{{\mathscr {M}}}}\right| -1\right) }{2}} {\textstyle \frac{2T\left( x_{I}^{\left( {{{\mathscr {M}}}}\right) } ,S_{i}^{n_{c} } \right) }{\left| {{{\mathscr {M}}}}\right| }} =\left( \left| {{{\mathscr {M}}}}\right| -1\right) T\left( x_{I}^{\left( {{{\mathscr {M}}}}\right) } ,S_{i}^{n_{c} } \right) , \end{aligned}$$

while (78) can be rewritten as

81 $$\begin{aligned} B\left( {{\mathscr {S}}}_{n_{c} } ,{{\mathscr {S}}}_{{\mathscr {N}}} \right) ={\textstyle \frac{\left| {{\mathscr {S}}}_{{\mathscr {N}}} \right| \left( \left| {{\mathscr {S}}}_{{\mathscr {N}}} \right| -1\right) }{2}} {\textstyle \frac{B\left( R_{q}^{\left( {{\mathscr {S}}}_{{\mathscr {N}}} \right) } ,S_{i}^{n_{c} } \right) }{\left| {{\mathscr {S}}}_{{\mathscr {N}}} \right| }} ={\textstyle \frac{\left( \left| {{\mathscr {S}}}_{{\mathscr {N}}} \right| -1\right) }{2}} B\left( R_{q}^{\left( {{\mathscr {S}}}_{{\mathscr {N}}} \right) } ,S_{i}^{n_{c} } \right) ; \end{aligned}$$

thus the ratio of (79) also follows.

As a corollary, using the total entanglement throughput $T\left( {{\mathscr {S}}}_{{\mathscr {N}}} \right)$ (16) of the entangled connections of ${{\mathscr {S}}}_{{\mathscr {N}}}$ (Bell states per *C*) and the total traffic $T\left( {{{\mathscr {M}}}}\right)$ of ${{{\mathscr {M}}}}$,

82 $$\begin{aligned} T\left( {{{\mathscr {M}}}}\right) =\left( \left| {{{\mathscr {M}}}}\right| -1\right) \left( \sum _{i=1}^{n_{c} }T\left( x_{i} \right) \right) , \end{aligned}$$

the ratio

83 $$\begin{aligned} \mu \left( T\left( {{\mathscr {S}}}_{{\mathscr {N}}} \right) ,T\left( {{{\mathscr {M}}}}\right) \right) ={\textstyle \frac{T\left( {{\mathscr {S}}}_{{\mathscr {N}}} \right) }{T\left( {{{\mathscr {M}}}}\right) }} ={\textstyle \frac{1}{2}} \end{aligned}$$

follows.

Therefore, with respect to the amount of total traffic, the proposed strongly-entangled network structure ${{\mathscr {S}}}_{{\mathscr {N}}}$ is two times more efficient than a classical full-mesh network structure ${{{\mathscr {M}}}}$.

The proof is concluded here. $\square$

### Random routing

#### Theorem 4

(Random routing efficiency via the strongly-entangled structure). *The structure* $${{\mathscr {S}}}_{{\mathscr {N}}} $$ *enables an efficient random routing for all the* $$n_{c} $$ *low-priority quantum repeaters* $$R_{i} $$, $$i=1,\ldots ,n_{c} ,$$ *via the total number of entanglement swapping operations* $$\left| U_{S} \left( {{\mathscr {S}}}_{{\mathscr {N}}} ,R_{i} \right) \right| $$ *in* $${{\mathscr {S}}}_{{\mathscr {N}}} $$ *for the serving of* $$R_{i} $$, *with* $$\Pr \left( \left| U_{S} \left( {{\mathscr {S}}}_{{\mathscr {N}}} ,R_{i} \right) \right| \ge 2c\right) \le {\textstyle \frac{1}{2^{\left| {{\mathscr {S}}}_{{\mathscr {N}}} \right| } }} ,$$* for any* $$c\ge 1$$.

#### *Proof*

Our aim here is to show that the probability that more than entanglement swapping operation is required in a particular uniform randomly selected $$R_{I}^{\left( {{\mathscr {S}}}_{{\mathscr {N}}} \right) } $$ ingress quantum repeater of $${{\mathscr {S}}}_{{\mathscr {N}}} $$ to construct the entangled path between the source $$R_{i} $$ and egress quantum repeater $$R_{E}^{\left( {{\mathscr {S}}}_{{\mathscr {N}}} \right) } $$ of $${{\mathscr {S}}}_{{\mathscr {N}}} $$ is low.

Let $\left| {{\mathscr {S}}}_{{\mathscr {N}}} \right|$ be the number of $R_{q}^{\left( {{\mathscr {S}}}_{{\mathscr {N}}} \right) }$ quantum repeaters, $q=1,\ldots ,\left| {{\mathscr {S}}}_{{\mathscr {N}}} \right|$ in the strongly-entangled structure ${{\mathscr {S}}}_{{\mathscr {N}}}$, and let $\left| E\left( {{\mathscr {S}}}_{{\mathscr {N}}} \right) \right|$ be the number of entangled connections within ${{\mathscr {S}}}_{{\mathscr {N}}}$, $\left| E\left( {{\mathscr {S}}}_{{\mathscr {N}}} \right) \right| ={\textstyle \frac{\left| {{\mathscr {S}}}_{{\mathscr {N}}} \right| \cdot \left( \left| {{\mathscr {S}}}_{{\mathscr {N}}} \right| -1\right) }{2}}$.

Then, let $$R_{i} $$ a source quantum node from the set $${{\mathscr {S}}}_{n_{c} } $$ of the $$n_{c} $$ low-priority quantum repeaters, $$\left| {{\mathscr {S}}}_{n_{c} } \right| =n_{c} $$. Then, a given $$R_{I}^{\left( {{\mathscr {S}}}_{{\mathscr {N}}} \right) } $$ ingress quantum repeater is selected for $$R_{i} $$ with probability

84 $$\begin{aligned} \Pr \left( R_{i} \rightarrow R_{I}^{\left( {{\mathscr {S}}}_{{\mathscr {N}}} \right) } \right) ={\textstyle \frac{1}{\left| {{\mathscr {S}}}_{{\mathscr {N}}} \right| }} , \end{aligned}$$

to formulate the random entangled path $${{\mathscr {P}}}_{i} $$ from $$R_{i} $$ to $$R_{I}^{\left( {{\mathscr {S}}}_{{\mathscr {N}}} \right) } $$,

85 $$\begin{aligned} {{\mathscr {P}}}_{i} ={{\mathscr {P}}}\left( R_{I}^{\left( {{\mathscr {S}}}_{{\mathscr {N}}} \right) } ,R_{i} \right) . \end{aligned}$$

Then, let assume that a random path $${{\mathscr {P}}}_{i} $$ requires the egress quantum repeater $$R_{E}^{\left( {{\mathscr {S}}}_{{\mathscr {N}}} \right) } $$, that formulates entangled path between $$R_{i} $$ and $$R_{I}^{\left( {{\mathscr {S}}}_{{\mathscr {N}}} \right) } $$

86 $$\begin{aligned} {{\mathscr {P}}}_{i} ={{\mathscr {P}}}\left( R_{E}^{\left( {{\mathscr {S}}}_{{\mathscr {N}}} \right) } ,R_{i} \right) . \end{aligned}$$

**Figure 5:**
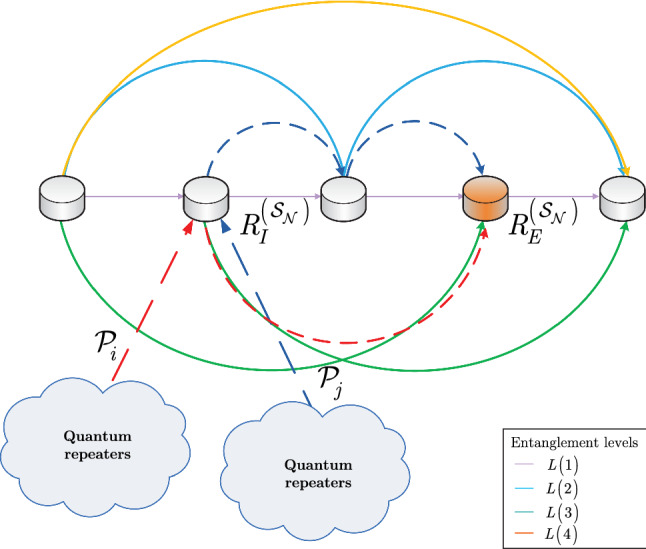
A collision of entangled paths in the strongly-entangled structure. The ingress and egress quantum repeaters associated with the paths within the strongly-entangled structure coincide. Entangled path ${{\mathscr {P}}}_{i}$ is depicted by a red dashed line, and entangled path ${{\mathscr {P}}}_{j}$ is depicted by a blue dashed line.

Let $f\left( \cdot \right)$ be an indicator function, defined as

87 $$\begin{aligned} f\left( {{\mathscr {P}}}_{i} ,{{\mathscr {P}}}_{j} \right) :=\left\{ \begin{array}{l} {1,{\mathrm{\; if\; }}\left( R_{I}^{\left( {{\mathscr {S}}}_{{\mathscr {N}}} \right) } \left( {{\mathscr {P}}}_{i} \right) =R_{I}^{\left( {{\mathscr {S}}}_{{\mathscr {N}}} \right) } \left( {{\mathscr {P}}}_{j} \right) \right) \wedge \left( R_{E}^{\left( {{\mathscr {S}}}_{{\mathscr {N}}} \right) } \left( {{\mathscr {P}}}_{i} \right) =R_{E}^{\left( {{\mathscr {S}}}_{{\mathscr {N}}} \right) } \left( {{\mathscr {P}}}_{j} \right) \right) } \\ {0,{\mathrm{\; otherwise}}} \end{array}\right. , \end{aligned}$$

where $R_{I}^{\left( {{\mathscr {S}}}_{{\mathscr {N}}} \right) } \left( {{\mathscr {P}}}_{x} \right) \in {{\mathscr {S}}}_{{\mathscr {N}}}$ and $R_{E}^{\left( {{\mathscr {S}}}_{{\mathscr {N}}} \right) } \left( {{\mathscr {P}}}_{x} \right) \in {{\mathscr {S}}}_{{\mathscr {N}}}$ are the ingress and egress quantum repeaters of path ${{\mathscr {P}}}_{x}$. Thus, the indicator function indicates an event if the $R_{I}^{\left( {{\mathscr {S}}}_{{\mathscr {N}}} \right) } \left( {{\mathscr {P}}}_{i} \right)$ ingress node of ${{\mathscr {P}}}_{i}$ coincidences with the $R_{I}^{\left( {{\mathscr {S}}}_{{\mathscr {N}}} \right) } \left( {{\mathscr {P}}}_{j} \right)$ ingress node of ${{\mathscr {P}}}_{j}$ and the $R_{E}^{\left( {{\mathscr {S}}}_{{\mathscr {N}}} \right) } \left( {{\mathscr {P}}}_{i} \right)$ egress node of ${{\mathscr {P}}}_{i}$ coincidences with the $R_{E}^{\left( {{\mathscr {S}}}_{{\mathscr {N}}} \right) } \left( {{\mathscr {P}}}_{j} \right)$ egress node of ${{\mathscr {P}}}_{j}$. Thus, a $f\left( {{\mathscr {P}}}_{i} ,{{\mathscr {P}}}_{j} \right) =1$ situation therefore indicates a collision between the paths ${{\mathscr {P}}}_{i}$ and ${{\mathscr {P}}}_{j}$ (A collision situation is illustrated in Fig. 5.).

Since for ${{\mathscr {P}}}_{i}$ and ${{\mathscr {P}}}_{j}$, the $R_{I}^{\left( {{\mathscr {S}}}_{{\mathscr {N}}} \right) }$ ingress quantum repeaters are selected independently and uniformly random within ${{\mathscr {S}}}_{n_{c} }$, it follows that for the entangled paths $\left\{ {{\mathscr {P}}}_{i} ,{{\mathscr {P}}}_{j} ,{{\mathscr {P}}}_{k} \right\}$, the $f\left( {{\mathscr {P}}}_{i} ,{{\mathscr {P}}}_{j} \right)$ and $f\left( {{\mathscr {P}}}_{i} ,{{\mathscr {P}}}_{k} \right)$ indicator functions are independent random variables for $i\ne j\ne k\ne i$.

As follows, indicator functions $f\left( {{\mathscr {P}}}_{i} ,{{\mathscr {P}}}_{j} \right)$ and $f\left( {{\mathscr {P}}}_{i} ,{{\mathscr {P}}}_{k} \right)$ can be rewritten as Bernoulli random variables

88 $$\begin{aligned} X_{j} =f\left( {{\mathscr {P}}}_{i} ,{{\mathscr {P}}}_{j} \right) \end{aligned}$$

and

89 $$\begin{aligned} X_{k} =f\left( {{\mathscr {P}}}_{i} ,{{\mathscr {P}}}_{k} \right) , \end{aligned}$$

such that

90 $$\begin{aligned} \Pr \left( X_{j} =1\right) ={{\mathbb {E}}}\left[ X_{j} \right] \le {\textstyle \frac{\left| {{\mathscr {P}}}_{{{\mathscr {S}}}_{{\mathscr {N}}} } \right| }{\left| E\left( {{\mathscr {S}}}_{{\mathscr {N}}} \right) \right| }} , \end{aligned}$$

where$\left| {{\mathscr {P}}}_{{{\mathscr {S}}}_{{\mathscr {N}}} } \right|$ is the path length within ${{\mathscr {S}}}_{{\mathscr {N}}}$, such that

91 $$\begin{aligned} \left| {{\mathscr {P}}}_{{{\mathscr {S}}}_{{\mathscr {N}}} } \right| =1 \end{aligned}$$

due to the structural attributes of ${{\mathscr {S}}}_{{\mathscr {N}}}$. (Thus, (91) holds because only one entangled connection within ${{\mathscr {S}}}_{{\mathscr {N}}}$ is required for the swapping from the ingress node to an egress node.).

As follows, (90) can be rewritten as

92 $$\begin{aligned} \Pr \left( X_{j} =1\right) ={{\mathbb {E}}}\left[ X_{j} \right] \le {\textstyle \frac{1}{{\textstyle \frac{\left| {{\mathscr {S}}}_{{\mathscr {N}}} \right| \cdot \left( \left| {{\mathscr {S}}}_{{\mathscr {N}}} \right| -1\right) }{2}} }} ={\textstyle \frac{2}{\left| {{\mathscr {S}}}_{{\mathscr {N}}} \right| \cdot \left( \left| {{\mathscr {S}}}_{{\mathscr {N}}} \right| -1\right) }} \end{aligned}$$

Taking (92) for all the $\left| {{\mathscr {S}}}_{{\mathscr {N}}} \right|$ nodes, yields a tail distribution for the sum of $\left| {{\mathscr {S}}}_{{\mathscr {N}}} \right|$ Bernoulli variables, as

93 $$\begin{aligned} \Pr \left( X_{\Sigma } \ge x\right) , \end{aligned}$$

where $X_{\Sigma }$ is the sum of $\left| {{\mathscr {S}}}_{{\mathscr {N}}} \right|$ Bernoulli random variables,

94 $$\begin{aligned} X_{\Sigma } =\sum _{j=1}^{\left| {{\mathscr {S}}}_{{\mathscr {N}}} \right| }X_{j} , \end{aligned}$$

for any positive *x*, with a relation by Markov inequality

95 $$\begin{aligned} \Pr \left( X_{\Sigma } \ge x\right) \le {\textstyle \frac{\left| {{\mathscr {S}}}_{{\mathscr {N}}} \right| {{\mathbb {E}}}\left[ X_{j} \right] }{x}} ={\textstyle \frac{\left| {{\mathscr {S}}}_{{\mathscr {N}}} \right| {\textstyle \frac{2}{\left| {{\mathscr {S}}}_{{\mathscr {N}}} \right| \cdot \left( \left| {{\mathscr {S}}}_{{\mathscr {N}}} \right| -1\right) }} }{x}} ={\textstyle \frac{2}{x\left( \left| {{\mathscr {S}}}_{{\mathscr {N}}} \right| -1\right) }} \le {\textstyle \frac{1}{x}} . \end{aligned}$$

Then, since (95) is not sufficiently small if

96 $$\begin{aligned} x=2c \end{aligned}$$

for any constant *c*, (95) can be reformulated as

97 $$\begin{aligned} \Pr \left( X_{\Sigma } \ge x\right) \le \Pr \left( e^{nX_{\Sigma } } \ge e^{nx} \right) \le {\textstyle \frac{{{\mathbb {E}}}\left[ e^{nX_{\Sigma } } \right] }{e^{nx} }} , \end{aligned}$$

for any positive *n*.

Thus, from the Chernoff-bound^181^, the relation

98 $$\begin{aligned} \Pr \left( X_{\Sigma } \ge x\right) \le \mathop {\min }\limits _{n>0} {\textstyle \frac{{{\mathbb {E}}}\left[ e^{nX_{\Sigma } } \right] }{e^{nx} }} , \end{aligned}$$

follows.

Since $X_{\Sigma }$ is the sum of $\left| {{\mathscr {S}}}_{{\mathscr {N}}} \right|$ Bernoulli random variables, ${{\mathbb {E}}}\left[ e^{nX_{\Sigma } } \right]$ can be evaluated as

99 $$\begin{aligned} \begin{aligned} {\mathbb {E}}\left[ {{e}^{n{{X}_{\Sigma }}}} \right]&={\mathbb {E}}\left[ {{e}^{n\sum \limits _{j=1}^{\left| {{\mathscr {S}}_{\mathscr {N}}} \right| }{{{X}_{j}}}}} \right] \\&=\prod \limits _{j=1}^{\left| {{\mathscr {S}}_{\mathscr {N}}} \right| }{{\mathbb {E}}\left[ {{e}^{n{{X}_{j}}}} \right] } \\&={{\left( {\mathbb {E}}\left[ {{e}^{n{{X}_{j}}}} \right] \right) }^{\left| {{\mathscr {S}}_{\mathscr {N}}} \right| }} \\&={{\left( p{{e}^{n}}+\left( 1-p \right) \right) }^{\left| {{\mathscr {S}}_{\mathscr {N}}} \right| }} \\&={{\left( 1+p\left( {{e}^{n}}-1 \right) \right) }^{\left| {{\mathscr {S}}_{\mathscr {N}}} \right| }}, \end{aligned} \end{aligned}$$

that can be rewritten as

100 $$\begin{aligned} {{\mathbb {E}}}\left[ e^{nX_{\Sigma } } \right] \le e^{\left( e^{n} -1\right) \left| {{\mathscr {S}}}_{{\mathscr {N}}} \right| {{\mathbb {E}}}\left[ X_{j} \right] } , \end{aligned}$$

since $1+a\le e^{a}$, by theory.

Therefore, $\Pr \left( X_{\Sigma } \ge x\right)$ can be rewritten as

101 $$\begin{aligned} \Pr \left( X_{\Sigma } \ge x\right) =\mathop {\min }\limits _{n>0} {\textstyle \frac{e^{\left( e^{n} -1\right) \left| {{\mathscr {S}}}_{{\mathscr {N}}} \right| {{\mathbb {E}}}\left[ X_{j} \right] } }{e^{nx} }} , \end{aligned}$$

thus at

102 $$\begin{aligned} n=\ln \left( 1+\xi \right) \end{aligned}$$

the following relation is yielded

103 $$\begin{aligned} \Pr \left( X_{\Sigma } \ge \left( 1+\xi \right) \left| {{\mathscr {S}}}_{{\mathscr {N}}} \right| {{\mathbb {E}}}\left[ X_{j} \right] \right) \le \left( {\textstyle \frac{e^{\xi } }{\left( 1+\xi \right) ^{1+\xi } }} \right) ^{\left| {{\mathscr {S}}}_{{\mathscr {N}}} \right| {{\mathbb {E}}}\left[ X_{j} \right] } . \end{aligned}$$

It can be verified, that if $\xi$ is sufficiently large, then (103) can be rewritten as

104 $$\begin{aligned} \Pr \left( X_{\Sigma } \ge c\left| {{\mathscr {S}}}_{{\mathscr {N}}} \right| {{\mathbb {E}}}\left[ X_{j} \right] \right) \le \left( {\textstyle \frac{1}{2}} \right) ^{\left| {{\mathscr {S}}}_{{\mathscr {N}}} \right| {{\mathbb {E}}}\left[ X_{j} \right] } , \end{aligned}$$

thus the probability that for a given resource node $$R_{i} $$ with path $${{\mathscr {P}}}_{i} $$ more than one $$U_{S} \left( {{\mathscr {S}}}_{{\mathscr {N}}} ,R_{i} \right) $$ entanglement swapping operation is required within $${{\mathscr {S}}}_{{\mathscr {N}}} $$ to construct the entangled path between $$R_{i} $$ and $$R_{E}^{\left( {{\mathscr {S}}}_{{\mathscr {N}}} \right) } \left( {{\mathscr {P}}}_{i} \right) $$, is yielded as

105 $$\begin{aligned} \Pr \left( \left| U_{S} \left( {{\mathscr {S}}}_{{\mathscr {N}}} ,R_{i} \right) \right| \ge 2c\right) \le {\textstyle \frac{1}{2^{\left| {{\mathscr {S}}}_{{\mathscr {N}}} \right| } }} , \end{aligned}$$

where $$c\ge 1$$ is a positive integer, while $$\left| U_{S} \left( {{\mathscr {S}}}_{{\mathscr {N}}} ,R_{i} \right) \right| $$ is the number of entanglement swapping operations within $${{\mathscr {S}}}_{{\mathscr {N}}} $$ associated with $$R_{i} $$.

The proof is therefore concluded here. $\square$

A path collision between entangled paths ${{\mathscr {P}}}_{i}$ and ${{\mathscr {P}}}_{j}$ in the strongly-entangled structure ${{\mathscr {S}}}_{{\mathscr {N}}}$ is illustrated in Fig. 5. Both entangled paths are associated with the same ingress node $R_{I}^{\left( {{\mathscr {S}}}_{{\mathscr {N}}} \right) } \left( {{\mathscr {P}}}_{i} \right) =R_{I}^{\left( {{\mathscr {S}}}_{{\mathscr {N}}} \right) } \left( {{\mathscr {P}}}_{j} \right)$ and egress node $R_{E}^{\left( {{\mathscr {S}}}_{{\mathscr {N}}} \right) } \left( {{\mathscr {P}}}_{i} \right) =R_{E}^{\left( {{\mathscr {S}}}_{{\mathscr {N}}} \right) } \left( {{\mathscr {P}}}_{j} \right)$. Assuming that $R_{I}^{\left( {{\mathscr {S}}}_{{\mathscr {N}}} \right) }$ and $R_{E}^{\left( {{\mathscr {S}}}_{{\mathscr {N}}} \right) }$ share only one entangled connection within ${{\mathscr {S}}}_{{\mathscr {N}}}$, only the serving of one path from $R_{I}^{\left( {{\mathscr {S}}}_{{\mathscr {N}}} \right) }$ to $R_{E}^{\left( {{\mathscr {S}}}_{{\mathscr {N}}} \right) }$ is allowed. Path ${{\mathscr {P}}}_{i}$ is served via the entangled connection between $R_{I}^{\left( {{\mathscr {S}}}_{{\mathscr {N}}} \right) }$ and $R_{E}^{\left( {{\mathscr {S}}}_{{\mathscr {N}}} \right) }$, while the serving of ${{\mathscr {P}}}_{j}$ is decomposed as $R_{I}^{\left( {{\mathscr {S}}}_{{\mathscr {N}}} \right) } \rightarrow R_{z}^{\left( {{\mathscr {S}}}_{{\mathscr {N}}} \right) } \rightarrow R_{E}^{\left( {{\mathscr {S}}}_{{\mathscr {N}}} \right) }$, where $R_{z}^{\left( {{\mathscr {S}}}_{{\mathscr {N}}} \right) } \ne R_{E}^{\left( {{\mathscr {S}}}_{{\mathscr {N}}} \right) }$.

### Fault tolerance

#### Theorem 5

(Fault tolerance of the strongly-entangled structure). *The strongly-entangled structure provides a seamless service at* $1\le k\le \left| {{\mathscr {S}}}_{{\mathscr {N}}} \right| -2$ *arbitrary entangled connection failures by increasing the entanglement throughputs of the remaining* $\left| E\left( {{\mathscr {S}}}_{{\mathscr {N}}} \right) \right| -k$ *entangled connections of* ${{\mathscr {S}}}_{{\mathscr {N}}}$.

#### *Proof*

Let $Q\left( R_{q}^{\left( {{\mathscr {S}}}_{{\mathscr {N}}} \right) } ,R_{z}^{\left( {{\mathscr {S}}}_{{\mathscr {N}}} \right) } \right) ={\textstyle \frac{1}{\left| {{\mathscr {S}}}_{{\mathscr {N}}} \right| }} B\left( R_{q}^{\left( {{\mathscr {S}}}_{{\mathscr {N}}} \right) } ,R_{i} \right)$ be the entanglement throughputs (see (50)) of the entangled connections within ${{\mathscr {S}}}_{{\mathscr {N}}}$ at no failures.

At *k* entangled connection failures, let $\Delta _{k}$ be the increment of the entanglement throughputs (Bell states per *C*) of the remaining $\left| E\left( {{\mathscr {S}}}_{{\mathscr {N}}} \right) \right| -k$ entangled connections of ${{\mathscr {S}}}_{{\mathscr {N}}}$, and let

106 $$\begin{aligned} Q_{k}^{*} \left( R_{q}^{\left( {{\mathscr {S}}}_{{\mathscr {N}}} \right) } ,R_{z}^{\left( {{\mathscr {S}}}_{{\mathscr {N}}} \right) } \right) =Q\left( R_{q}^{\left( {{\mathscr {S}}}_{{\mathscr {N}}} \right) } ,R_{z}^{\left( {{\mathscr {S}}}_{{\mathscr {N}}} \right) } \right) +\Delta _{k} \end{aligned}$$

be the updated entanglement throughputs of the entangled connections of ${{\mathscr {S}}}_{{\mathscr {N}}}$ (Bell states per *C*).

Let us define entangled connection failure events $\mathrm{E}_{1}$, $\mathrm{E}_{2}$ and $\mathrm{E}_{3}$ in the following manner:

107 $$\begin{aligned} {\mathrm{E}}:=\left\{ \begin{array}{l} {{\mathrm{E}}_{1} {\mathrm{,\; if\; }}k=1} \\ {{\mathrm{E}}_{2} {\mathrm{,\; if\; }}\left( k=2\right) \vee \left( k=\left| {{\mathscr {S}}}_{{\mathscr {N}}} \right| -2\right) \vee \left( \left| {{\mathscr {S}}}_{{\mathscr {N}}} \right| \le 6\right) } \\ {{\mathrm{E}}_{3} {\mathrm{,\; otherwise}}.} \end{array}\right. \end{aligned}$$

Then, using the formalisms of^136^, after some calculations $\Delta _{k}$ can be evaluated as

108 $$\begin{aligned} \Delta _{k} =\left\{ \begin{array}{l} {{\textstyle \frac{1}{2}} \left( {\textstyle \frac{1}{\left| {{\mathscr {S}}}_{{\mathscr {N}}} \right| -2}} B\left( R_{q}^{\left( {{\mathscr {S}}}_{{\mathscr {N}}} \right) } ,{{\mathscr {S}}}_{n_{c} } \right) +{\textstyle \frac{1}{\left| {{\mathscr {S}}}_{{\mathscr {N}}} \right| }} B\left( R_{q}^{\left( {{\mathscr {S}}}_{{\mathscr {N}}} \right) } ,{{\mathscr {S}}}_{n_{c} } \right) \right) -{\textstyle \frac{1}{\left| {{\mathscr {S}}}_{{\mathscr {N}}} \right| }} B\left( R_{q}^{\left( {{\mathscr {S}}}_{{\mathscr {N}}} \right) } ,{{\mathscr {S}}}_{n_{c} } \right) ,\mathrm{\; if\; E}=\mathrm{E}_{1} } \\ {{\textstyle \frac{1}{2}} \left( {\textstyle \frac{1}{\left| {{\mathscr {S}}}_{{\mathscr {N}}} \right| -k-1}} B\left( R_{q}^{\left( {{\mathscr {S}}}_{{\mathscr {N}}} \right) } ,{{\mathscr {S}}}_{n_{c} } \right) +{\textstyle \frac{1}{\left| {{\mathscr {S}}}_{{\mathscr {N}}} \right| -1}} B\left( R_{q}^{\left( {{\mathscr {S}}}_{{\mathscr {N}}} \right) } ,{{\mathscr {S}}}_{n_{c} } \right) \right) -{\textstyle \frac{1}{\left| {{\mathscr {S}}}_{{\mathscr {N}}} \right| }} B\left( R_{q}^{\left( {{\mathscr {S}}}_{{\mathscr {N}}} \right) } ,{{\mathscr {S}}}_{n_{c} } \right) \mathrm{,\; if\; E}=\mathrm{E}_{2} } \\ {\left( {\textstyle \frac{1}{\left| {{\mathscr {S}}}_{{\mathscr {N}}} \right| -k}} B\left( R_{q}^{\left( {{\mathscr {S}}}_{{\mathscr {N}}} \right) } ,{{\mathscr {S}}}_{n_{c} } \right) \right) -{\textstyle \frac{1}{\left| {{\mathscr {S}}}_{{\mathscr {N}}} \right| }} B\left( R_{q}^{\left( {{\mathscr {S}}}_{{\mathscr {N}}} \right) } ,{{\mathscr {S}}}_{n_{c} } \right) \mathrm{,\; if\; E}=\mathrm{E}_{3} .} \end{array}\right. \end{aligned}$$

As follows, at an initial $Q\left( R_{q}^{\left( {{\mathscr {S}}}_{{\mathscr {N}}} \right) } ,R_{z}^{\left( {{\mathscr {S}}}_{{\mathscr {N}}} \right) } \right) ={\textstyle \frac{1}{\left| {{\mathscr {S}}}_{{\mathscr {N}}} \right| }} B\left( R_{q}^{\left( {{\mathscr {S}}}_{{\mathscr {N}}} \right) } ,R_{i} \right)$, the updated $Q_{k}^{*} \left( R_{q}^{\left( {{\mathscr {S}}}_{{\mathscr {N}}} \right) } ,R_{z}^{\left( {{\mathscr {S}}}_{{\mathscr {N}}} \right) } \right)$ at the failure of *k* entangled connections in ${{\mathscr {S}}}_{{\mathscr {N}}}$ is on the order of

109 $$\begin{aligned} Q_{k}^{*} \left( R_{q}^{\left( {{\mathscr {S}}}_{{\mathscr {N}}} \right) } ,R_{z}^{\left( {{\mathscr {S}}}_{{\mathscr {N}}} \right) } \right) \cong {\textstyle \frac{1}{\left| {{\mathscr {S}}}_{{\mathscr {N}}} \right| -k}} B\left( R_{q}^{\left( {{\mathscr {S}}}_{{\mathscr {N}}} \right) } ,{{\mathscr {S}}}_{n_{c} } \right) . \end{aligned}$$

As *h* quantum repeater $R_{q}^{\left( {{\mathscr {S}}}_{{\mathscr {N}}} \right) }$ fails within ${{\mathscr {S}}}_{{\mathscr {N}}}$, then the structure of ${{\mathscr {S}}}_{{\mathscr {N}}}$ becomes a strongly-entangled network formulated by $\left| {{\mathscr {S}}}_{{\mathscr {N}}} \right| -h$ quantum repeaters, therefore $\Delta _{h}$ is yielded as

110 $$\begin{aligned} \Delta _{h} ={\textstyle \frac{1}{\left| {{\mathscr {S}}}_{{\mathscr {N}}} \right| -k}} B\left( R_{q}^{\left( {{\mathscr {S}}}_{{\mathscr {N}}} \right) } ,{{\mathscr {S}}}_{n_{c} } \right) -{\textstyle \frac{1}{\left| {{\mathscr {S}}}_{{\mathscr {N}}} \right| }} B\left( R_{q}^{\left( {{\mathscr {S}}}_{{\mathscr {N}}} \right) } ,{{\mathscr {S}}}_{n_{c} } \right) . \end{aligned}$$

If both *k* entangled connections and *h* quantum repeater $R_{q}^{\left( {{\mathscr {S}}}_{{\mathscr {N}}} \right) }$ fails in the structure, then the problem is analogous to *k* entangled connection failures within a strongly-entangled structure formulated by $\left| {{\mathscr {S}}}_{{\mathscr {N}}} \right| -h$ quantum repeaters^135,136^. Therefore, $\Delta _{k,h}$ can be evaluated via (108) and (110) in the following manner:

111 $$\begin{aligned} \Delta _{k,h} =\left\{ \begin{array}{l} {{\textstyle \frac{1}{2}} \left( {\textstyle \frac{1}{\left( \left| {{\mathscr {S}}}_{{\mathscr {N}}} \right| -h\right) -2}} B\left( R_{q}^{\left( {{\mathscr {S}}}_{{\mathscr {N}}} \right) } ,{{\mathscr {S}}}_{n_{c} } \right) +{\textstyle \frac{1}{\left| {{\mathscr {S}}}_{{\mathscr {N}}} \right| -h}} B\left( R_{q}^{\left( {{\mathscr {S}}}_{{\mathscr {N}}} \right) } ,{{\mathscr {S}}}_{n_{c} } \right) \right) -{\textstyle \frac{1}{\left| {{\mathscr {S}}}_{{\mathscr {N}}} \right| }} B\left( R_{q}^{\left( {{\mathscr {S}}}_{{\mathscr {N}}} \right) } ,{{\mathscr {S}}}_{n_{c} } \right) ,\mathrm{\; if\; E}=\mathrm{E}_{1} } \\ {{\textstyle \frac{1}{2}} \left( {\textstyle \frac{1}{\left( \left| {{\mathscr {S}}}_{{\mathscr {N}}} \right| -h\right) -k-1}} B\left( R_{q}^{\left( {{\mathscr {S}}}_{{\mathscr {N}}} \right) } ,{{\mathscr {S}}}_{n_{c} } \right) +{\textstyle \frac{1}{\left( \left| {{\mathscr {S}}}_{{\mathscr {N}}} \right| -h\right) -1}} B\left( R_{q}^{\left( {{\mathscr {S}}}_{{\mathscr {N}}} \right) } ,{{\mathscr {S}}}_{n_{c} } \right) \right) -{\textstyle \frac{1}{\left| {{\mathscr {S}}}_{{\mathscr {N}}} \right| }} B\left( R_{q}^{\left( {{\mathscr {S}}}_{{\mathscr {N}}} \right) } ,{{\mathscr {S}}}_{n_{c} } \right) \mathrm{,\; if\; E}=\mathrm{E}_{2} } \\ {\left( {\textstyle \frac{1}{\left( \left| {{\mathscr {S}}}_{{\mathscr {N}}} \right| -h\right) -k}} B\left( R_{q}^{\left( {{\mathscr {S}}}_{{\mathscr {N}}} \right) } ,{{\mathscr {S}}}_{n_{c} } \right) \right) -{\textstyle \frac{1}{\left| {{\mathscr {S}}}_{{\mathscr {N}}} \right| }} B\left( R_{q}^{\left( {{\mathscr {S}}}_{{\mathscr {N}}} \right) } ,{{\mathscr {S}}}_{n_{c} } \right) \mathrm{,\; if\; E}=\mathrm{E}_{3} .} \end{array}\right. \end{aligned}$$

$$\square $$

## Performance evaluation

Here, we analyze the performance of the strongly-entangled structure ${{\mathscr {S}}}_{{\mathscr {N}}}$ and compare it with a classical full-mesh structure ${{{\mathscr {M}}}}$. Using the results of “Method” and “Strongly-entangled structure for resource balancing in the quantum internet” sections, a numerical evidence is given to characterize the amount of transmitted traffic within the structures as a function of the number of nodes, to characterize the fanout coefficients of the structures as a function of the number of nodes, and to compare the traffic increments of the connections at connection failures. For the comparison between classical resource balancing and quantum resource balancing, the results of “Method” and “Strongly-entangled structure for resource balancing in the quantum internet” sections are compared with the results of^135,136^.

**Figure 6:**
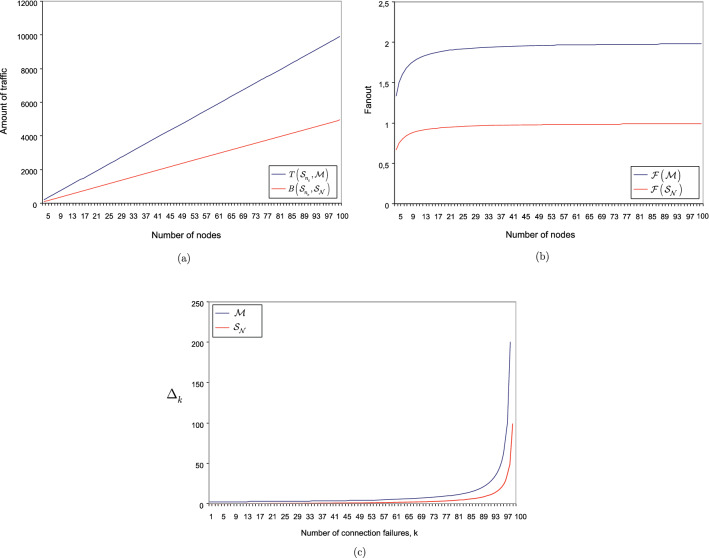
(**a**) Comparison of the amounts of traffic $T\left( {{\mathscr {S}}}_{n_{c} } ,{{{\mathscr {M}}}}\right)$ and $B\left( {{\mathscr {S}}}_{n_{c} } ,{{\mathscr {S}}}_{{\mathscr {N}}} \right)$ (Bell states per *C*) within the structures of ${{{\mathscr {M}}}}$ and ${{\mathscr {S}}}_{{\mathscr {N}}}$, with $T\left( x_{I}^{\left( {{{\mathscr {M}}}}\right) } ,S_{i}^{n_{c} } \right) =B\left( R_{q}^{\left( {{\mathscr {S}}}_{{\mathscr {N}}} \right) } ,S_{i}^{n_{c} } \right) =100$ and $\left| {{{\mathscr {M}}}}\right| =\left| {{\mathscr {S}}}_{{\mathscr {N}}} \right| =1,\ldots ,100$. (**b**) Comparison of the fanout coefficients ${{{\mathscr {F}}}}\left( {{{\mathscr {M}}}}\right)$ and ${{{\mathscr {F}}}}\left( {{\mathscr {S}}}_{{\mathscr {N}}} \right)$ of the structures of ${{{\mathscr {M}}}}$ and ${{\mathscr {S}}}_{{\mathscr {N}}}$, with $\left| {{{\mathscr {M}}}}\right| =\left| {{\mathscr {S}}}_{{\mathscr {N}}} \right| =1,\ldots ,100$. (**c**) The entanglement throughput increment $\Delta _{k}$ (Bell states per *C*) of the $\left| E\left( {{\mathscr {S}}}_{{\mathscr {N}}} \right) \right| -k$ entangled connections at the failure of *k* entangled connections within ${{\mathscr {S}}}_{{\mathscr {N}}}$, with $k=1,\ldots ,100$, $\left| {{\mathscr {S}}}_{{\mathscr {N}}} \right| =100$, $B\left( R_{q}^{\left( {{\mathscr {S}}}_{{\mathscr {N}}} \right) } ,R_{i} \right) =100$, and $Q\left( R_{q}^{\left( {{\mathscr {S}}}_{{\mathscr {N}}} \right) } ,R_{z}^{\left( {{\mathscr {S}}}_{{\mathscr {N}}} \right) } \right) ={\textstyle \frac{1}{\left| {{\mathscr {S}}}_{{\mathscr {N}}} \right| }} B\left( R_{q}^{\left( {{\mathscr {S}}}_{{\mathscr {N}}} \right) } ,R_{i} \right) =1$, $Q_{k}^{*} \left( R_{q}^{\left( {{\mathscr {S}}}_{{\mathscr {N}}} \right) } ,R_{z}^{\left( {{\mathscr {S}}}_{{\mathscr {N}}} \right) } \right) \cong {\textstyle \frac{1}{\left| {{\mathscr {S}}}_{{\mathscr {N}}} \right| -k}} B\left( R_{q}^{\left( {{\mathscr {S}}}_{{\mathscr {N}}} \right) } ,{{\mathscr {S}}}_{n_{c} } \right)$. For the comparison between classical resource balancing and quantum resource balancing, the proposed results are compared with the results of^135,136^.

In Fig. 6a the amounts of traffic are compared within a strongly-entangled structure ${{\mathscr {S}}}_{{\mathscr {N}}}$ and a classical full-mesh structure ${{{\mathscr {M}}}}$. In Fig. 6b, the fanout coefficients of the structures are compared. In Fig. 6c compares the fault tolerant capabilities of the structures.

The strongly-entangled quantum network is two times more effective than a classical full-mesh structure: The required amount of traffic is half that of the classical structure^135,136^, the fanout coefficient of the strongly-entangled structure is half that of the classical structure, and the required entanglement throughput of the entangled connection is half that of the classical structure. As future work, our aim is to provide a detailed performance comparison with other related approaches on resource allocation and routing in quantum networks^62,70,71^.

## Conclusions

Here, we defined methods and procedures for optimizing the resource allocation mechanisms of the quantum Internet. We proposed a model for resource consumption optimization of quantum repeaters, proposed a method for optimizing the entanglement swapping procedure, and studied the conditions of deadlock-free entanglement swapping. We defined a strongly-entangled network structure for optimal resource balancing in the quantum Internet. We proved the resource-balancing efficiency of the strongly-entangled structure and its fault tolerance.

## Supplementary information

Supplementary material 1.

## Data Availability

This work does not have any experimental data.
