# Supplementary material for: Resource prioritization and balancing for the quantum internet
Source: Sci Rep. 2020 Dec 28;10:22390. doi: 10.1038/s41598-020-78960-5 (PMC7770047; doi:10.1038/s41598-020-78960-5)
Supplement: Supplementary file 1 — Supplementary material 1. [file 41598_2020_78960_MOESM1_ESM.pdf]

Resource Prioritization and Balancing for the Quantum Internet

Laszlo Gyongyosi[1,2,3,*] **and Sandor Imre**[2]

[1]School of Electronics and Computer Science, University of Southampton, Southampton, SO17 1BJ, UK
[2]Department of Networked Systems and Services, Budapest University of Technology and Economics, Budapest, H-1117 Hungary
[3]MTA-BME Information Systems Research Group, Hungarian Academy of Sciences, Budapest, H-1051 Hungary
[*]gyongyosi@hit.bme.hu

ABSTRACT

The quantum Internet enables networking based on the fundamentals of quantum mechanics. Here, methods and procedures of resource prioritization and resource balancing are defined for the quantum Internet. We define a model for resource consumption optimization in quantum repeaters, and a strongly-entangled network structure for resource balancing. We study the resource-balancing efficiency of the strongly-entangled structure. We prove that a strongly-entangled quantum network is two times more efficient in a resource balancing problem than a full-mesh network of the traditional Internet.

A Appendix

A.1 Deadlock-Free Entanglement Swapping

Here, we show that there exists a $\mathrm{P}(N)$ pre-processing method for N that yields a deadlock-free entanglement swapping for all quantum repeaters of N.

The $\mathrm{P}(N)$ pre-processing method for N is defined in Procedure A.1.

The input of Sub-Procedure A.1 is a set $\mathscr{S}_{\mathscr{P}}(\rho,A,B)$ of available paths in N for a particular ρ is a density matrix between a source A and destination B. The output of Sub-Procedure A.1 is a set $\mathscr{S}'_{\mathscr{P}}(\rho,A,B)$ of deadlock-free paths for the U_S entanglement swapping.

The motivation behind the maximization of $f_{\mathrm{P}(N)}$ in (A.6) is as follows. Because the ratio $\left|\mathscr{S}'_{\mathscr{P}}(\rho,A,B)\right| / \left|\mathscr{S}_{\mathscr{P}}(\rho,A,B)\right|$ is high, the transmission has a high flexibility since the number of available quantum repeaters is also high. As a corollary, the resource consumption $\tilde{\mathscr{C}}(R_i)$ of a given quantum repeater R_i can be decreased. A density ρ with a higher expected fidelity $\mathbb{E}(F(\rho))$ requires a higher flexibility and resource consumption in the transmission procedure; therefore, $\mathbb{E}(F(\rho))$ is a weighting coefficient in $f_{\mathrm{P}(N)}$.

A.1.1 Computational Complexity

The computational complexity of $\mathrm{P}(N)$ for the deadlock-free entanglement swapping in N, for $\mathscr{S}_{\varphi}(N)$ dependencies and with a set of $\mathscr{S}_{\varphi}(\mathrm{L}_l)$ entangled connections in the dependency relations, is

$$\mathscr{O}\left(\left|\mathscr{S}_{\varphi}(N)\right| + \left|\mathscr{S}_{\varphi}(\mathrm{L}_l)\right|\right), \tag{A.7}$$

due to a depth-first search[1–5] in the channel dependency graph of N.

A.2 Notations

The notations of the manuscript are summarized in Table A.1.

Table A.1. Summary of notations.

Notation	*Description*
N	An entangled quantum network, $N=(V,E)$, where V is a set of nodes, E is a set of entangled connections.
A	A source user (quantum node) in the quantum network.

B	A destination user (quantum node).
R_i	An i-th quantum repeater, $i = 1, \ldots, q$, where q is the total number of quantum repeaters.
R_h	A previous neighbor of R_i.
R_j	A next neighbor of R_i (towards destination).
l	Level of entanglement.
$\mathrm{L}_l(x,y)$	An l-level entangled connection between quantum nodes x and y.
$d(x,y)_{\mathrm{L}_l}$	Hop-distance at an L_l-level entangled connection between quantum nodes x and y, $d(x,y)_{\mathrm{L}_l} = 2^{l-1}$.
O_C	An oscillator with frequency f_C, $f_C = 1/t_C$, serves as a reference clock.
C	A cycle, with $t_C = 1/f_C$.
q	Total number of quantum repeaters in an entangled path $\mathscr{P}(A_i \to B_i)$, $q = d(A,B)_{\mathrm{L}_l} - 1$.
B_F	Entanglement throughput [Bell states per C].
$\lvert B_F \rvert$	Number of entangled states [Number of Bell states].
$\mathscr{C}(R_i)$	Computational consumption in a quantum repeater R_i.
$\mathrm{L}_l(k)$	A k-th entangled connection.
$B_F(\mathrm{L}_l(k))$	Entanglement throughput of the entangled connection $\mathrm{L}_l(k)$ [Bell states per C].
$\alpha(R_i, \mathrm{L}_l(k))$	Computational consumption in quantum repeater R_i associated with a k-th entangled connection $\mathrm{L}_l(k)$, $k = 1, \ldots, z$, where z is the total number of entangled connections of R_i.
$\Upsilon(R_i, \mathrm{L}_l(k))$	Computational consumption in quantum repeater R_i associated with the quantum memory usage at $\mathrm{L}_l(k)$.
$\phi(R_i, \mathrm{L}_l(k))$	Computational consumption in quantum repeater R_i associated with the entanglement purification of $\mathrm{L}_l(k)$.
$\tau(R_i, \mathrm{L}_l(k))$	Computational consumption in quantum repeater R_i associated with the entanglement distribution towards a target node B.
$\nu(R_i, \mathrm{L}_l(k))$	Computational consumption in quantum repeater R_i associated with the entanglement swapping of $\mathrm{L}_l(k)$.
$\zeta(R_i, \mathrm{L}_l(k))$	Computational consumption in quantum repeater R_i associated with the path selection.
$C(R_i, \mathrm{L}_l(k))$	Cost of auxiliary classical communications in quantum repeater R_i.
$\mathscr{C}(R_i)$	Total computational consumption in quantum repeater R_i.
$\chi(R_i)$	Total number of received entangled states [Number of Bell states].
$\lvert B_F(A) \rvert$	Number of entangled states outputted by a source node A [Number of Bell states].
$\mathscr{S}_{\mathscr{P}}(R_i)$	Set of $\mathscr{P}$ paths pass through quantum repeater R_i.
$\mathscr{P}_s(x,y)$	An s-th path between quantum nodes x and y, $s = 1, \ldots, \lvert \mathscr{S}_{xy} \rvert$, where $\mathscr{S}_{xy}$ is the set of paths between x and y, $\lvert \mathscr{S}_{xy} \rvert$ is the cardinality of $\mathscr{S}_{xy}$.

$\omega(\mathscr{P}_s)$	Weight of entanglement throughput set to the s-th path $\mathscr{P}_s$, $\omega(\mathscr{P}_s) \in [0,1]$, $s = 1,\ldots,\lvert\mathscr{S}_{AB}\rvert$, such that for a particular source and target pair (A,B) of $\mathscr{P}_s$, $\sum_{s=1}^{\lvert\mathscr{S}_{AB}\rvert} \omega(\mathscr{P}_s(A,B)) = 1$.
$f(\mathscr{C})$	Objective function subject to a minimization.
$\Omega(R_i)$	Constraint.
$B_F^*(R_i,R_j)$	Upper bound on the entanglement throughput between quantum repeaters R_i and R_j connected by the entangled connection $\mathrm{L}_l(R_i,R_j)$ [Bell states per C].
$\Gamma(\mathrm{L}_l(R_i,R_j))$	Constraint.
$\mathscr{S}_{\mathscr{P}}(N)$	Set of entangled paths, $\mathscr{S}_{\mathscr{P}}(N) = \{\mathscr{P}_1,\ldots,\mathscr{P}_n\}$, where $\mathscr{P}_i$ is an i-th entangled path.
$\phi(\mathscr{P}_i)$	Weighted entanglement throughput of the path [Bell states per C].
$D(\omega_s(x,y))$	An optimal distribution of the weights that minimizes objective function $f(\mathscr{C})$.
$\xi(N)$	Maximal weighted entanglement throughputs of the paths in $\mathscr{S}_{\mathscr{P}}(N)$ [Bell states per C].
$\nu(N)$	Minimal weighted entanglement throughputs of the paths in $\mathscr{S}_{\mathscr{P}}(N)$ [Bell states per C].
$\mu(N)$	Difference of $\xi(N)$ and $\nu(N)$, $\mu(N) = \lvert\xi(N) - \nu(N)\rvert$ [Bell states per C].
$\mu^*(N)$	Target value for $\mu(N)$ [Bell states per C].
$D(\cdot)$	A randomization operator.
$\phi'(\mathscr{P}_i)$	Updated weighted entanglement throughput of path $\mathscr{P}_i$ [Bell states per C].
$\Delta(\phi(\mathscr{P}_i))$	An upper bound on the difference $\lvert\phi'(\mathscr{P}_i) - \phi(\mathscr{P}_i)\rvert$ [Bell states per C].
$\mathscr{C}'(R_i)$	Updated value of $\mathscr{C}(R_i)$.
$f^*(\mathscr{C})$	A desired objective function value.
$\min(\tilde{\mathscr{C}}^*(R_i))$	A target value of the maximal computational consumption $\mathscr{C}^*(R_i)$ of quantum repeater R_i.
$\mathscr{S}_D$	Set of updated weights.
U_S	Entanglement swapping operator.
ρ_A	Input entangled density matrix (i.e., a half pair of a Bell state).
$\mathscr{A}(\rho_A)$	Set of possible outcoming entangled states in R_i.
$\beta(\rho_A)$	Subsystem ρ_A of the entangled state β_{AB}.
$\beta(\sigma_B)$	Subsystem σ_B of the entangled state β_{AB}.
$R_s(\beta(\rho_A))$	A source quantum node with $\beta(\rho_A)$.
$R_d(\beta(\sigma_B))$	A destination quantum node with $\beta(\sigma_B)$.
$\mathscr{D}(R_i)$	Set of R_d destination quantum nodes that share an entangled connection with a current quantum repeater R_i.
$\mathscr{S}_{\mathscr{P}}(R_i,\rho_A)$	Set of entangled connections that contains the entangled connection via an entanglement swapping in a particular quantum repeater R_i, using input state ρ_A and output state σ_B.
$\mathscr{Q}(\sigma_{B,i})$	Source set of g input entangled states for a given $\sigma_{B,i}$.

$\mathrm{P}(N)$	Pre-processing method for N.
$\mathscr{S}_{\mathscr{P}}(N)$	Set of n entangled paths subject to be formulated in N, $\mathscr{S}_{\mathscr{P}}(N) = \{\mathscr{P}_1, \ldots, \mathscr{P}_n\}$.
$\lvert\phi'(\mathscr{S}_{\mathscr{P}}(N))\rvert$	Total number of entangled states subject to be transmitted over the n entangled paths [Number of Bell states].
$\varphi(\mathrm{L}_l(x,y), \mathrm{L}_l(y,z))$	A dependency $\mathrm{L}_l(x,y) \to \mathrm{L}_l(y,z)$ of entangled connections $\mathrm{L}_l(x,y)$ and $\mathrm{L}_l(y,z)$ between quantum nodes (x,y) and (y,z).
$\mathrm{X}_{\mathscr{P}}$	A set of paths, $\mathrm{X}_{\mathscr{P}}(\varphi(\mathrm{L}_l(x,y), \mathrm{L}_l(y,z)))$, that contains the dependency $\varphi(\mathrm{L}_l(x,y), \mathrm{L}_l(y,z))$.
$\mathscr{S}_{\mathscr{P}}(\rho, A, B)$	A set of available paths with cardinality $\lvert\mathscr{S}_{\mathscr{P}}(\rho, A, B)\rvert$ for a given density matrix ρ between a source A and destination B.
$\mathscr{P}(\rho)$	A path for ρ between a source A and destination B.
$\Pi(\rho)$	Set of connection dependencies with respect to ρ, subject to be deleted via $\mathrm{P}(N)$.
$F(\rho)$	Fidelity of density ρ.
$\mathscr{S}_{\varphi}(N)$	Number of dependencies in N.
$\mathscr{S}_{\varphi}(\mathrm{L}_l)$	Set of entangled connections in the dependency relations.
R_i	A low-priority quantum node with a non-served resource request.
$B(R_i)$	Total entanglement throughput request of R_i [Bell states per C].
$D(R_i)$	Destination node of R_i.
$\mathscr{S}_{\mathscr{N}}$	A strongly-entangled quantum network.
$\lvert\mathscr{S}_{\mathscr{N}}\rvert$	Number of quantum repeaters in $\mathscr{S}_{\mathscr{N}}$.
$\lvert E(\mathscr{S}_{\mathscr{N}})\rvert$	Total number of entangled connections within $\mathscr{S}_{\mathscr{N}}$.
$\mathscr{M}$	A classical full-mesh network.
n_c	Number of low-priority quantum nodes (quantum nodes with non-servable resource requests) in N.
$n_{\mathscr{P}}$	Number of parallel entangled paths $\mathscr{P}\left(R_q^{(\mathscr{S}_{\mathscr{N}})}, R_i\right)$ between the quantum repeaters of $\mathscr{S}_{\mathscr{N}}$ and R_i, $n_{\mathscr{P}} = \lvert\mathscr{S}_{\mathscr{N}}\rvert$.
$n_{\Sigma\mathscr{P}}$	Total number of parallel entangled connections established via the $\mathscr{S}_{\mathscr{N}}$ structure at n_c low-priority quantum nodes, $n_{\Sigma\mathscr{P}} = n_c \lvert\mathscr{S}_{\mathscr{N}}\rvert$.
$L(d(x,y))$	Entangled connections in function of the $d(x,y)$ hop-distance between quantum nodes $\{x,y\} \in \mathscr{S}_{\mathscr{N}}$ in the $\mathscr{S}_{\mathscr{N}}$ strongly-entangled structure.
$R_I^{(\mathscr{S}_{\mathscr{N}})}$	Ingress quantum repeater of $\mathscr{S}_{\mathscr{N}}$.
$R_E^{(\mathscr{S}_{\mathscr{N}})}$	Egress quantum repeater of $\mathscr{S}_{\mathscr{N}}$.
$R_q^{(\mathscr{S}_{\mathscr{N}})}$	An q-th quantum repeater of $\mathscr{S}_{\mathscr{N}}$, $q = 1, \ldots, \lvert\mathscr{S}_{\mathscr{N}}\rvert$.
$B\left(R_i^{(\mathscr{S}_{\mathscr{N}})}, R_i\right)$	An entanglement throughput request from the low-priority node R_i [Bell states per C].
$Q\left(R_i^{(\mathscr{S}_{\mathscr{N}})}, R_q^{(\mathscr{S}_{\mathscr{N}})}\right)$	Entanglement throughput between quantum repeaters $R_i^{(\mathscr{S}_{\mathscr{N}})}$ and $R_q^{(\mathscr{S}_{\mathscr{N}})}$ of $\mathscr{S}_{\mathscr{N}}$ [Bell states per C].
$B(\mathscr{S}_{\mathscr{N}})$	Total entanglement throughput requests from the n_c low-priority quantum repeaters to $\mathscr{S}_{\mathscr{N}}$, $B(\mathscr{S}_{\mathscr{N}}) = \sum_{q=1}^{\lvert\mathscr{S}_{\mathscr{N}}\rvert} B\left(R_q^{(\mathscr{S}_{\mathscr{N}})}, \mathscr{S}_{n_c}\right) = \sum_{i=1}^{n_c} B(R_i)$ [Bell states per C].

$\mathscr{R}_{\mathscr{S}}$	Set of random quantum repeaters.
$\mathscr{R}\left(R_i^{(\mathscr{S}_\mathscr{N})}, R_i\right)$	Set of random nodes between $R_i^{(\mathscr{S}_\mathscr{N})}$ and R_i.
$U_S\left(R_q^{(\mathscr{S}_\mathscr{N})}\right)$	Entanglement swapping operation applied in $R_q^{(\mathscr{S}_\mathscr{N})}$ of $\mathscr{S}_\mathscr{N}$.
$B_F\left(\mathscr{P}\left(R_i, R_E^{(\mathscr{S}_\mathscr{N})}\right)\right)$	Entanglement throughput of entangled path $\mathscr{P}_j\left(R_i, R_E^{(\mathscr{S}_\mathscr{N})}\right)$ [Bell states per C].
$\mathscr{S}_{n_c}$	A set of n_c low-priority quantum repeaters.
$W\left(R_q^{(\mathscr{S}_\mathscr{N})}\right)$	Total entanglement throughput associated with $R_q^{(\mathscr{S}_\mathscr{N})}$ within the structure of $\mathscr{S}_\mathscr{N}$ [Bell states per C].
$Z\left(\mathscr{S}_\mathscr{N}\right)$	Cumulated entanglement throughput of the quantum repeaters of $\mathscr{S}_\mathscr{N}$ [Bell states per C].
$T\left(\mathscr{S}_\mathscr{N}\right)$	Total entanglement throughput of the entangled connections of $\mathscr{S}_\mathscr{N}$ [Bell states per C].
$\mathscr{F}\left(R_q^{(\mathscr{S}_\mathscr{N})}\right)$	Fanout coefficient, ratio of the $W\left(R_q^{(\mathscr{S}_\mathscr{N})}\right)$ total entanglement throughput of entangled connections within $\mathscr{S}_\mathscr{N}$ and the $B\left(R_q^{(\mathscr{S}_\mathscr{N})}, \mathscr{S}_{n_c}\right)$ incoming request from the low-priority quantum repeaters.
$\mathscr{F}\left(\mathscr{S}_\mathscr{N}\right)$	Fanout of $\mathscr{S}_\mathscr{N}$, the maximum fanout among the quantum repeaters of $\mathscr{S}_\mathscr{N}$.
$\mu\left(x,y\right)$	Ratio of x and y, $\mu\left(x,y\right) = \frac{x}{y}$.
$B\left(\mathscr{S}_{n_c}, \mathscr{S}_\mathscr{N}\right)$	Total traffic in $\mathscr{S}_\mathscr{N}$ at $\left|\mathscr{S}_\mathscr{N}\right|$ quantum repeaters and a set $\mathscr{S}_{n_c}$ of low-priority quantum repeaters.
$\left|U_S\left(\mathscr{S}_\mathscr{N}, R_i\right)\right|$	Total number of entanglement swapping operations in $\mathscr{S}_\mathscr{N}$ for the serving of R_i.
c	Constant.
$\Pr\left(R_i \to R_I^{(\mathscr{S}_\mathscr{N})}\right)$	Probability that a $R_I^{(\mathscr{S}_\mathscr{N})}$ ingress quantum repeater is selected for the serving of R_i.
$f\left(\cdot\right)$	Indicator function.
$R_I^{(\mathscr{S}_\mathscr{N})}\left(\mathscr{P}_x\right)$	Ingress quantum repeater of path $\mathscr{P}_x$, $R_I^{(\mathscr{S}_\mathscr{N})}\left(\mathscr{P}_x\right) \in \mathscr{S}_\mathscr{N}$.
$R_E^{(\mathscr{S}_\mathscr{N})}\left(\mathscr{P}_x\right)$	Egress quantum repeaters of path $\mathscr{P}_x$, $R_E^{(\mathscr{S}_\mathscr{N})}\left(\mathscr{P}_x\right) \in \mathscr{S}_\mathscr{N}$.
X_Σ	Sum of $\left|\mathscr{S}_\mathscr{N}\right|$ Bernoulli random variables.
k	Number of entangled connection failures within $\mathscr{S}_\mathscr{N}$.
h	Number of quantum repeater failures within $\mathscr{S}_\mathscr{N}$.
Δ_k	Increment of the entanglement throughputs of $\left|E\left(\mathscr{S}_\mathscr{N}\right)\right| - k$ entangled connections of $\mathscr{S}_\mathscr{N}$ at the failure of k entangled connections [Bell states per C].
E	An event.
$\Delta_{k,h}$	Increment of the entanglement throughputs of $\left|E\left(\mathscr{S}_\mathscr{N}\right)\right| - k$ entangled connections of $\mathscr{S}_\mathscr{N}$, at the failures of k entangled connections and h quantum repeaters [Bell states per C].

References

1. Qian, Z. and Tsui, C.Y. A Thermal Aware Routing Algorithm for Application-Specific Network-on-Chip. In: Palesi, M. and Daneshtalab, M. (Editors) *Routing Algorithms in Networks-on-Chip*, Springer, ISBN 978-1-4614-8273-4, ISBN 978-1-4614-8274-1 (eBook) (2014).

Procedure A.1 Deadlock-free entanglement swapping

Input: A set $\mathscr{S}_{\mathscr{P}}(N) = \{\mathscr{P}_1, \ldots, \mathscr{P}_n\}$ of n entangled paths in N, where $\mathscr{P}_s(A,B)$ is an s-th path, $s = 1, \ldots, n$, with A and B source and target quantum nodes on an s-th entangled path $\mathscr{P}_s$. Quantity $|\phi'(\mathscr{S}_{\mathscr{P}}(N))|$: number of entangled density matrices transmitted over the n entangled paths.
Output: Set $\mathscr{S}'_{\mathscr{P}}(\rho, A, B)$ of deadlock-free paths for the U_S entanglement swapping, $\forall \rho \in \mathscr{S}_{\mathscr{P}}(N)$.

Step 1. Let N be a quantum network with a set of R_i, $i = 1, \ldots, q$ quantum repeaters and a set $\mathscr{S}_{\mathscr{P}}(N) = \{\mathscr{P}_1, \ldots, \mathscr{P}_n\}$ of n entangled paths subject to be formulated in N, and let $|\phi'(\mathscr{S}_{\mathscr{P}}(N))|$ be the total number of entangled density matrices subject to be transmitted over the n entangled paths,

$$\left|\phi'(\mathscr{S}_{\mathscr{P}}(N))\right| = \sum_{i=1}^{n} \left|\phi'(\mathscr{P}_i)\right|. \tag{A.1}$$

Step 2. Let $\varphi(\mathrm{L}_l(x,y), \mathrm{L}_l(y,z))$ refer to a dependency $\mathrm{L}_l(x,y) \to \mathrm{L}_l(y,z)$ of entangled connections $\mathrm{L}_l(x,y)$ and $\mathrm{L}_l(y,z)$ between quantum nodes (x,y) and (y,z), and let $\mathrm{X}_{\mathscr{P}}(\varphi(\mathrm{L}_l(x,y), \mathrm{L}_l(y,z)))$ be the set of paths that contains the dependency $\varphi(\mathrm{L}_l(x,y), \mathrm{L}_l(y,z))$, as

$$\mathrm{X}_{\mathscr{P}}(\varphi(\mathrm{L}_l(x,y), \mathrm{L}_l(y,z))) = \{\mathscr{P}_i | \mathscr{P}_i \in \mathscr{S}_{\mathscr{P}}(N) \wedge \varphi(\mathrm{L}_l(x,y), \mathrm{L}_l(y,z))\}_i. \tag{A.2}$$

Step 3. For a given density matrix ρ between a source A and destination B, let $\mathscr{S}_{\mathscr{P}}(\rho, A, B)$ be the set of available paths with cardinality $|\mathscr{S}_{\mathscr{P}}(\rho, A, B)|$. Apply Sub-Procedure 1 to output a set $\mathscr{S}'_{\mathscr{P}}(\rho, A, B)$ of deadlock-free paths for ρ.
Step 4. Let $\mathscr{S}'_{\mathscr{P}}(\rho, A, B)$ be the set of available paths after Sub-Procedure 1, as

$$\mathscr{S}'_{\mathscr{P}}(\rho, A, B) = \mathscr{S}_{\mathscr{P}}(\rho, A, B) \setminus \mathscr{X}_{\mathscr{P}}(\rho, A, B), \tag{A.3}$$

where, for $\forall \varphi(L_l(x,y), L_l(y,z)) \in \Pi(\rho)$

$$\mathscr{X}_{\mathscr{P}}(\rho, A, B) = (\mathscr{P}(\rho) | \mathscr{P}(\rho) \in \mathscr{S}_{\mathscr{P}}(\rho, A, B)) \wedge (\mathscr{P}(\rho) \in \mathrm{X}_{\mathscr{P}}(\varphi(\mathrm{L}_l(x,y), \mathrm{L}_l(y,z)))). \tag{A.4}$$

where $\mathscr{P}(\rho)$ is a path for ρ between a source A and destination B, $\Pi(\rho)$ is the set of connection dependencies with respect to ρ, subject to be deleted via $\mathrm{P}(N)$. Set $\mathscr{S}'_{\mathscr{P}}(\rho, A, B)$ has a cardinality $\left|\mathscr{S}'_{\mathscr{P}}(\rho, A, B)\right|$, such that

$$|\mathscr{S}_{\mathscr{P}}(\rho, A, B)| \geq \left|\mathscr{S}'_{\mathscr{P}}(\rho, A, B)\right| \geq 1. \tag{A.5}$$

Step 5. Repeat steps 2-4 for all of the $|\phi'(\mathscr{S}_{\mathscr{P}}(N))|$ density matrices.
Step 6. Output $\mathscr{S}'_{\mathscr{P}}(\rho, A, B)$ for $\forall \rho \in \mathscr{S}_{\mathscr{P}}(N)$.

Sub-procedure A.1 Method of $\mathrm{P}(N)$

Input: Set $\mathscr{S}_{\mathscr{P}}(\rho, A, B)$ of available paths in N, where ρ is a density matrix between a source A and destination B.
Output: Set $\mathscr{S}'_{\mathscr{P}}(\rho, A, B)$ of deadlock-free paths for the U_S entanglement swapping.

Step 1. Define an objective function $f_{\mathrm{P}(N)}$ for method $\mathrm{P}(N)$ as

$$f_{\mathrm{P}(N)} = \max \frac{1}{|\phi'(\mathscr{S}_{\mathscr{P}}(N))|} \sum_{\rho \in |\phi'(\mathscr{S}_{\mathscr{P}}(N))|} \frac{\mathbb{E}(F(\rho)) \left|\mathscr{S}'_{\mathscr{P}}(\rho, A, B)\right|}{|\mathscr{S}_{\mathscr{P}}(\rho, A, B)|}, \tag{A.6}$$

where $\mathbb{E}(F(\rho))$ is the expected fidelity of ρ.
Step 2. Apply a depth-first search algorithm[1–3] for finding the strongly connected (a directed graph is called strongly connected if there is a path from each vertex to every other) components in the channel dependency graph[1,3] of N.
Step 3. Using the strongly connected components, eliminate the dependencies such that the objective function $f_{\mathrm{P}(N)}$ is maximized.
Step 4. Output the set $\mathscr{S}'_{\mathscr{P}}(\rho, A, B)$ of deadlock-free paths for the entanglement swapping.

2. Palesi, M., Holsmark, R., Kumar, S. and Catania, V. Application specific routing algorithms for networks on chip. *IEEE Trans. Parallel Distrib. Syst.* 20(3), 316–330 (2009).

3. Tarjan, R. Depth-first search and linear graph algorithms. *SIAM J. Comput.* 1(2), 146–160 (1972).

4. Duato, J. A necessary and sufficient condition for deadlock-free adaptive routing in wormhole networks. *IEEE Trans. Parallel Distrib. Syst.* 6(10), 1055–1067 (1995).

5. Chen, K. C., Chao, C. H., Lin, S. Y. and Wu, A. Y. Traffic- and Thermal-Aware Routing Algorithms for 3D Network-on-Chip (3D NoC) Systems. In: Palesi, M. and Daneshtalab, M. (Editors) *Routing Algorithms in Networks-on-Chip*, Springer, ISBN 978-1-4614-8273-4, ISBN 978-1-4614-8274-1 (eBook) (2014).
